# Leaf Trait-Environment Relationships in a Subtropical Broadleaved Forest in South-East China

**DOI:** 10.1371/journal.pone.0035742

**Published:** 2012-04-23

**Authors:** Wenzel Kröber, Martin Böhnke, Erik Welk, Christian Wirth, Helge Bruelheide

**Affiliations:** 1 Institute of Biology/Geobotany and Botanical Garden, Martin Luther University Halle Wittenberg, Halle, Germany; 2 Institute of Biology I, Special Botany and Functional Biodiversity Research, University of Leipzig, Leipzig, Germany; Norwegian University of Science and Technology, Norway

## Abstract

Although trait analyses have become more important in community ecology, trait-environment correlations have rarely been studied along successional gradients. We asked which environmental variables had the strongest impact on intraspecific and interspecific trait variation in the community and which traits were most responsive to the environment. We established a series of plots in a secondary forest in the Chinese subtropics, stratified by successional stages that were defined by the time elapsed since the last logging activities. On a total of 27 plots all woody plants were recorded and a set of individuals of every species was analysed for leaf traits, resulting in a trait matrix of 26 leaf traits for 122 species. A Fourth Corner Analysis revealed that the mean values of many leaf traits were tightly related to the successional gradient. Most shifts in traits followed the leaf economics spectrum with decreasing specific leaf area and leaf nutrient contents with successional time. Beside succession, few additional environmental variables resulted in significant trait relationships, such as soil moisture and soil C and N content as well as topographical variables. Not all traits were related to the leaf economics spectrum, and thus, to the successional gradient, such as stomata size and density. By comparing different permutation models in the Fourth Corner Analysis, we found that the trait-environment link was based more on the association of species with the environment than of the communities with species traits. The strong species-environment association was brought about by a clear gradient in species composition along the succession series, while communities were not well differentiated in mean trait composition. In contrast, intraspecific trait variation did not show close environmental relationships. The study confirmed the role of environmental trait filtering in subtropical forests, with traits associated with the leaf economics spectrum being the most responsive ones.

## Introduction

In recent years, community ecology has made much progress in understanding how the trait composition in a community changes along environmental gradients [1]–[3]. Still, predictions of trait-environment relationships are not straight forward because trait composition in a community is influenced by two opposing mechanisms [4]. On the one hand, the species in a community have to cope with the abiotic environmental setting (e.g. resource supply, disturbance etc.), resulting in abiotic environmental filtering of certain trait values [2], [5]–[7]. On the other hand, the species have to be sufficiently different in their niches, and thus also in the traits that reflect the niches, to avoid competitive exclusion [8]–[10].

As a result of environmental filtering, mean values of a trait will differ among communities along an environmental gradient, while, as a result of competitive exclusion, trait value distribution within communities will be divergent. Despite their alleged opposition, the two mechanisms are intimately linked in real communities, because the same trait can contribute to both niche segregation and competitive ability [11]. Then, environmental filtering might select for a trait that at the same time confers competitive superiority or the ability to facilitate other members of the community [12]. In this case, it might be considered to broaden the definition of environmental trait filtering and to lump together abiotic environmental filters and those brought about by biotic interactions [11]. Such a trait filter would also comply with the suggestion to model biotic interactions as a milieu or biotic background with which an organism interacts [13]. Including biotic interactions in the analysis of environmental filtering is particularly important for our study system, a successional series in a subtropical forest in China. Previous analyses from the study region have revealed that many environmental variables, such as soil pH, or topographical variables, such as aspect and slope, did not covary with successional stage, which led to the conclusion that the sampled forests have not been predominantly shaped by abiotic conditions but by biotic processes [14]. Thus, we also included successional stage and variables related to species richness among the environmental predictor variables, however, considering them as proxy variables without a causal relationship. Nevertheless, some abiotic environmental factors were found to covary with successional age, such as soil carbon, nitrogen content and soil moisture [14], patterns that have also been described from other succession series [15], [16]. Surprisingly few studies have attempted to relate shift in community mean trait values to environmental changes along forest succession series. From global trait relationships to soil fertility [6], [17], trait filtering would be expected to result in an increase in specific leaf area (SLA), leaf nitrogen content (LNC) and leaf phosphorous content (LPC) with increasing soil N and P supply. A typical forest succession series also reflects a strong gradient in light availability [18]. Light supply is also affected by elevation, inclination and aspect [19], which might vary independently of successional gradients. It is clear that behind these topographical variables there are more direct environmental drivers for plant performance such as UV radiation, temperature or air humidity [20]. However, elevation, inclination and aspect can be considered proxy variables for these unmeasured environmental variables that are difficult to assess in a field study.

A wide range of traits has been analysed on woody plants [21]–[23]. Among all characteristics, leaf traits are easily measured in comparison to other traits and offer a consistent basis for comparisons across a large range of plant life forms [1], [2], [24]. More importantly, many leaf traits are related to growth rates, as for example species with leaves with high SLA and LNC tend to have high nutrient concentrations and mass-based photosynthesis rates [2], [25]. In contrast, leaves with low SLA are physically more robust and less prone to herbivory and tend to live longer [26], [27]. In evergreen species, a low SLA is also associated with a higher shade tolerance [28]. In subtropical forests, SLA also reflects the difference between evergreen and deciduous leaf phenology type. Leaf nutrient concentration tend to vary positively with SLA, such as Mn [29] and S [30], as do heavy metals such as Pb [31]. Potassium, phosphorous and nitrogen contents have been found to limit tree growth in tropical forests [32], and thus, their leaf contents might be indicators of the plants' nutrient status. In addition, some cations such as calcium (Ca) might reflect transpiration, as Ca cannot be retranslocated from ageing leaves, and thus, Ca content might be taken as proxy for RGR and photosynthesis capacity (A_max_) for leaves of the same age. In contrast, hardly anything is known about many other leaf traits. For example, stomata density is generally thought to be positively related to a plant's ability to regulate transpiration, with higher densities of stomata associated with xeric morphology [33], [34]. Stomata density has been widely used to deduce atmospheric CO_2_ concentrations from fossilized leaves [35], but has also been described to increase with altitude [36].

While trait-environment relationships have already studied in species-rich subtropical forest ecosystems [37], [38], not much effort has been put so far into further dissecting the underlying link between traits and environment (but see [39]). It is unclear to which degree intraspecific trait variability does modulate species responses to environmental gradients. Although intraspecific trait variability across different habitats has been found to contribute less than mean trait values of different constituent species [40], this component cannot be neglected. Interspecific trait variation results in strong trait-environment relationships when (a) the range of trait values within communities is small and the community mean values are evenly dispersed along the trait gradient, or when (b) the spread of environmental values within species is small (narrow niche breadth) and species mean values are evenly dispersed along the environmental gradient (see simulation studies in [41]). The relationship becomes weaker when the spread (i.e. the variance) of values within communities or species increases and the means are clumped, i.e. do not cover the respective gradients. For example, a trait-environment relationship would still emerge with a high spread of trait values within communities but narrow niche breadths of species that are evenly dispersed along the environmental gradient. Conversely, a relationship would emerge in communities that are well separated along trait gradients but show broad niche breadths of species. Both mechanisms have to act in concert to result in environmental trait filtering. Still, one of the two might be more important than the other. Knowledge on these two components of the trait-environment link gives us insights into community assembly rules, as they inform us how well traits differentiate the different communities and how well the environmental gradient is covered by the species' niches. As we found broad niche breadths of species in our studied subtropical succession series [14], we expected only a weak link between species abundances and the environment. Because of the low degree of niche segregation of species along environmental gradients the community assembly in these forests can also be well explained by neutral models [42], [43]. As many tree species in this type of subtropical forest do occur in all age stages along the succession [44], [45], the secondary succession is best described by the pathway of initial floristic composition [14], [46]. In consequence, if there were any trait-environment relationships at all in these forests, they had to be brought about by a strong differentiation of communities in trait space.

**Figure 1:**
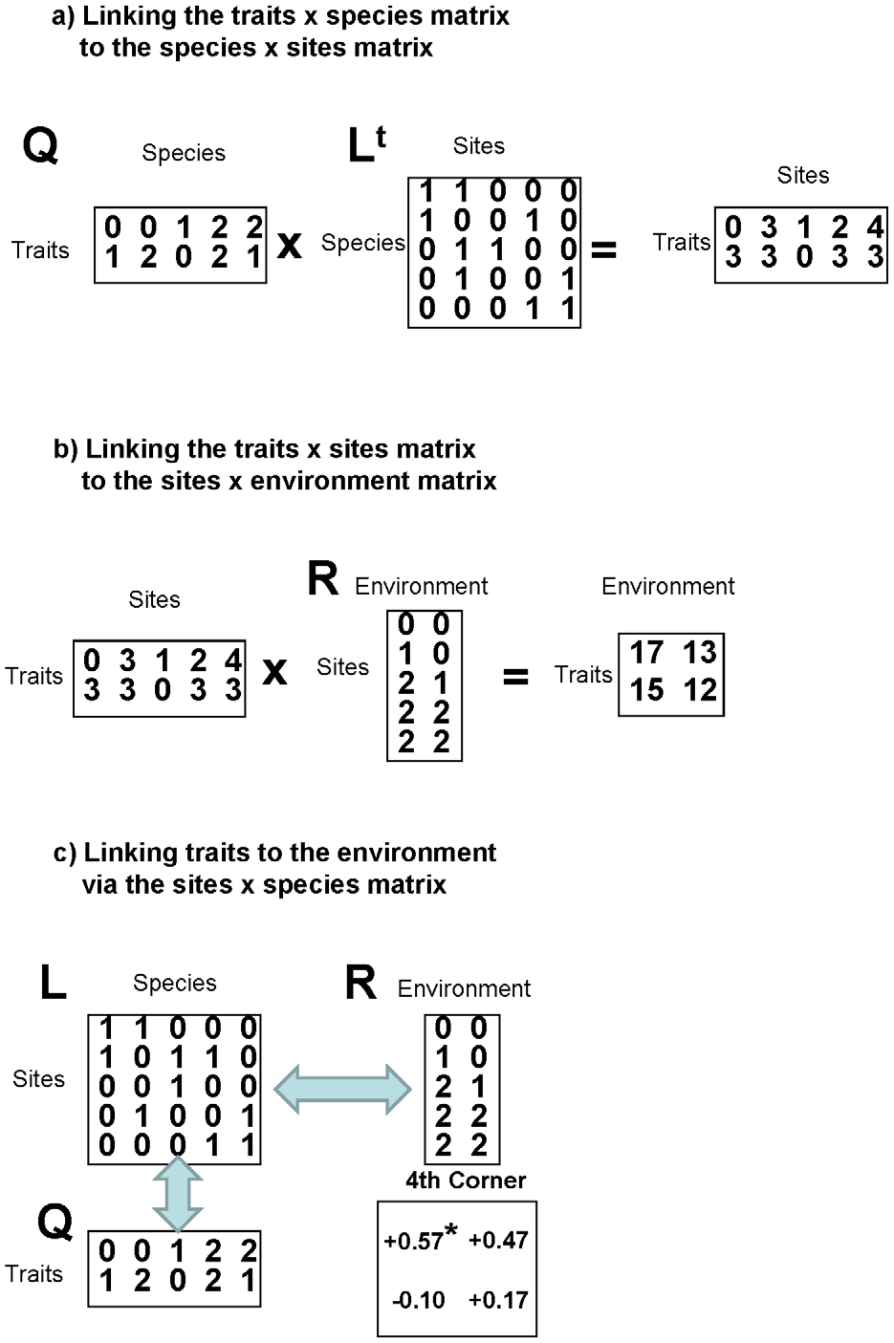
Scheme of linking traits to the environment in the Fourth Corner Analysis. The general procedure is shown by two matrix operations with an arbitrary data set. a: Multiplication of the traits×species matrix Q with the transposed species×sites matrixL, resulting in a traits×site matrix; b: Multiplication of the traits×sites matrix with the sites×environment matrix R, resulting in a traits×environment matrix; c: combining a and b in the Fourth Corner Analysis, showing Pearson correlation coefficients for the resulting in a traits×environment matrix significances derived from Model type I (see Fig. 2). Note that matrix multiplication satisfies the associativity rule, thus rendering it irrelevant whether first calculating the matrix product **QL** ^t^ or **L** ^t^ **R**. Furthermore, in contrast to this example, Fourth Corner Analysis does not use absolute abundances of species but fractions of total abundance in **L** and standardized values in **R** and **Q**. Moreover, the Fourth Corner algorithm does not use original matrix but inflated matrices where every non-empty entry or every occurrence in **L** becomes a new row (see [41]).

**Figure 2:**
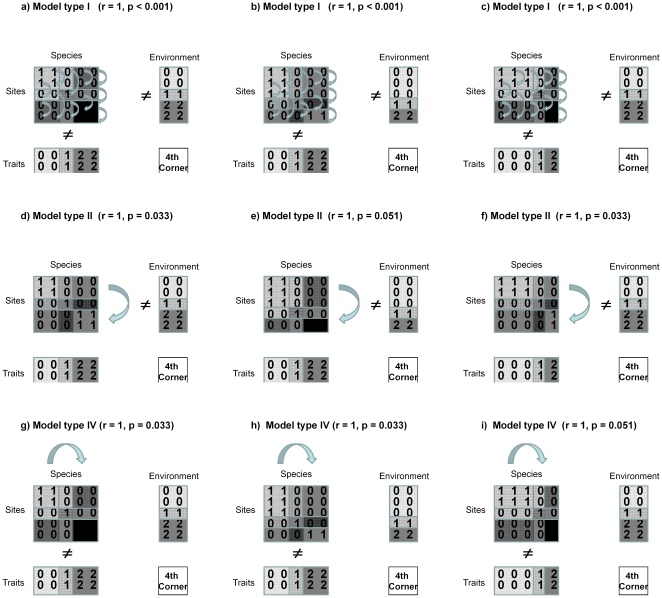
Scheme of the effect of different permutation methods in the Fourth Corner Analysis. The simplified test data set consists of a R-matrix of 5 sites×2 environmental variables, a L matrix of 5 sites×5 species and a Q matrix of 2 traits×5 species. The three columns show different proportions of environments and traits that reflect different heterogeneity in environment or traits, respectively. The left column shows equal variance between environment and traits, the middle column a skewed distribution of environmental variables and the right column a skewed distribution of traits. ‘≠’ indicates that link between the two matrices located aside the sign has been removed. The curved arrows show what is permutated in the L matrix, abundances within species (model type I), rows (type II) or columns (type IV). a, b, c: Model type I, reshuffling abundances for each species independently (i.e., within each column of the L matrix); thereby removing both the link between site and environment and the link between traits and species; d, e, f: Model type II, reshuffling rows of the L matrix, thereby removing the link between site and environment; g, h, i: Model type IV, reshuffling columns of the L matrix, thereby removing the link between traits and species. All permutations were repeated 99999 times. Please note that all models and proportions result in a perfect trait-environment correlation (r = 1), but differ in probability.

A tool to shed light on the relative importance of the two processes is provided by Fourth Corner Analysis [41]. Fourth Corner Analysis tests for correlations between traits and environment by linking a site×environment matrix (R) via a site×species abundance matrix (L) to a trait×species matrix (Q). Using an arbitrary example data set, Fig. 1a and b demonstrates that the trait-environment link can be conceived as two sequential matrix multiplications. Assessing the relative importance of each of these two matrix operations would allow conclusions as to whether environmental trait filtering depends more on the first or the second step. Appropriate tools to this approach are different permutation models, as provided by [41]. The different way of permuting abundances within columns or permuting whole rows and colums of the L matrix removes either the link between sites and the environment or between species and traits or both. Fig. 2 gives a schematic overview of these different permutation models, using a small idealized data set. Different proportions in environmental conditions realized across sites or in traits realized across species result in different degrees of significances obtained by reshuffling, depending on distribution of trait values within and among communities and of environmental values within and among species. The less well dispersed the environmental variable in model type II or the trait values in model type IV, the lower the levels of significance, seen in insignificant relationships in the two fictitious datasets in Fig. 2e and i.

We applied this analysis approach to a succession series in subtropical China, which had been established in the framework of the recently established biodiversity and ecosystem functioning experiment in subtropical China (BEF-China) [14]. We hypothesized that 1) most trait-environment relationships are encountered with environmental variables that covary with the successional gradient, such as soil carbon, nitrogen content and soil moisture, while environmental variables representing elevation, soil pH, aspect and slope are much less related to leaf traits;2) the most responsive leaf traits are those that reflect the leaf economics spectrum, i.e. those traits that covary with the worldwide gradient in chemical, structural and physiological leaf traits, which spans from species with long leaf lifetimes, high leaf construction costs (low SLA) and low nutrient concentrations (low LNC) to species with short leaf lifetimes, low dry-mass investment per leaf area and high leaf nutrient concentrations [2]; 3) trait-environment relationships are mostly brought by interspecific trait variation and to a much lesser degree by intraspecific trait variation; and 4) the association of communities with species leaf traits (the link between the Q and L matrix) is more important for explaining the encountered trait-environment relationships than the association of species with the environment (the link between the R and L matrix), which would indicate a high differentiation of communities in trait space.

## Materials and Methods

### Study site

The study was carried out in the Gutian Mountains (Gutianshan), a National Nature Reserve (NNR) situated near to the border triangle of the three provinces Zhejiang, Jiangxi and Fujian, about 30 km from the county capital Kaihua (China, 29°8′18″–29°17′29″N, 118°2′14″–118°11′12″E) [14]. It has an area of 8107 ha, and covers an elevational gradient between 250 m and 1258 m a.s.l. The Gutianshan NNR has a typical Chinese subtropical climate with an annual average precipitation ranging between 1793 mm and 1960 mm (109 cells in the Worldclim dataset with 30″ resolution [47]). Taking a mean lapse rate of 0.55°C/100 m [48], the local mean annual temperature of 15.3°C measured in the NNR at 440 m a.s.l. [49] and published long term data for the climate station data of Qu Xian (118.87°E, 28.97°N, 66.1 m a.s.l.), which is only about 75 km away from the study site, we estimated a mean annual temperature range between 16.3°C to 10.8°C for the whole NNR and between 16.3°C to 12.7°C between our plots (see below) at the lowest and highest altitude, respectively. Correspondingly, mean January plot temperatures range between 4.3°C and 0.9°C and mean July plot temperatures between 28.1°C and 24.4°C. The area is located in one of the global hot spots of phytodiversity [50], with 1426 seed-plant species of 648 genera and 149 families occurring in the reserve. Regarding the very high pressure of land use commonly found in China, this forest has been remarkably preserved from anthropogenic impacts. Part of the reserve has been under agricultural use, as evidenced in the occurrences of historic terraces in some of the plots. In addition, there are still illegal logging activities in low elevation part of the NNR, in addition to natural disturbances such as typhoon damages and snow and ice breakages. While old-growth stands cover a large part of the forest, with maximum tree age of about 180 years [51], younger successional stages occur as well. It is a mixed evergreen broadleaved forest with *Schima superba* (Gardn. et Champ). and *Castanopsis eyrei* (Champ. ex Benth.) Tutch. being the dominant species and almost even proportions between evergreen and deciduous species in terms of species number [52]. The prevailing soil type is Cambisol with a generally sandy-loamy texture. Soil depth varies between shallow soils less than 50 cm and deeply developed soils of more than 120 cm thickness. The stone content can exceed 90%-Vol at some locations in the deeper soil horizons. The soil parent rock is mainly granite or deeply weathered granite (saprolite).

### Study design

A set of 27 Comparative Study Plots (CSPs) was established in the Nature Reserve in summer 2008. The CSPs were stratified by successional stage, based on the guess of tree age and on local knowledge of the most recent logging event, and randomly selected within the following age classes (1: <20 yrs, 2: <40 yrs, 3: <60 yrs, 4: <80 yrs, 5: ≥80 yrs). Classification into successional stages was then confirmed by measurements of diameter at breast height (dbh) of all trees in a plot with dbh >10 cm and by tree age analyses based on stem cores. Tree age of the fifth largest tree in the plot corresponded to the age classes given above. Further details on plot selection and age class determination are given in [14].

On every plot, a complete inventory of individuals taller than one meter was carried out on an area of 30 m×30 m and a set of environmental variables was measured (elevation, slope, aspect, soil moisture, pH, C, N and C/N ratio) as well as structural variables (height and cover of layers). Elevation ranged from 251 m to 903 m a.s.l. Aspect was expressed as northness and eastness, using cosine and sine of aspect, respectively. Soil samples were taken from nine positions in every CSP in five different depth intervals (0–5, 5–10, 10–20, 20–30, and 30–50 cm). Analyses were carried out on air-dried and sieved (2 mm) samples, pooled per depth interval. However, in the present study only the uppermost horizon was used because it showed the largest variation in soil conditions. Soil pH was analysed potentiometrically in a 1∶2.5 soil-H_2_O solution. Total soil C and N were analysed with Vario ELIII elementar analyzer. All samples were non-calcareous, thus total C content equals organic carbon (C_org_). Soil moisture was analysed gravimetrically in campaigns extending only over a few days in June/July 2008, November 2008 and March 2009, and averaged over all dates. Soil water content was determined on 5 g soil after drying at 105°C for 24 h. Photosynthetically active radiation and red∶far-red ratio were measured at 1.3 m above the ground in nine subplots perplot, using a Licor LI-190SA PAR quantum sensor and a Skye Red/Far-Red sensor, respectively. Relative light intensity was expressed as ratio to a second PAR sensor outside the forest.

### Leaf sampling and leaf traits

Controlling for the profound effects of sun exposure on many leaf traits [53], leaf samples were taken from sun exposed branches with an expandable pruner. As some canopies reached 30 m and more, a minority (less than 10%) of all leaf samples were not completely sun exposed. Only completely developed leaves were sampled, without signs of herbivore damage and preferably free of leaf fungi. Leaf trait analysis followed the protocols for standardised trait measurements [54]. Seven leaves were taken per individual and stored in wet PVC bags until weighing in the field lab. Leaf area was obtained by scanning the fresh leaves and analysing the digital data with Win FOLIA Pro S (Regent Instruments Inc.). Two additional leaves were sampled and stored in 70% ethanol for stomata analysis. Stomatal density was assessed on both leaf surfaces, thus two enumerations per replicate, using the Zeiss microscope Axioskop 2 plus. Only one species had hyperstomatous leaves (*Alangium kurzii* Craib). Stomata were counted on a minimum of 50000 µm^2^. In addition, length and width were measured of three stomata per replicate. The area occupied by stomata was calculated from stomata density and assuming an ellipsoid shape of the stomata. For the majority of the species, samples were taken from one randomly chosen individual each from five to seven CSPs (for exact sample sizes see Appendix Table S1). Exceptions were those species that occurred in less than five plots (34 species). Species were mostly sampled with one individual per plot, with the exception of 30 rare species that were sampled with at maximum two individuals in one plot to obtain sufficient number of replicates. To reduce the effect of extreme values of leaf aluminium content, this variable was log transformed.

In total, traits were obtained from 416 individuals, representing 82% of all (148) species occurring in the 27 CSPs, thus providing a trait value for 95.2% of all (16120) individuals. Of the 773 individuals of species for which no trait value was obtained, 75% were conifers (mainly *Pinus massoniana*Lamb. and *Cunninghamia lanceolata* (Lamb.) Hook.). However, the occurrence and abundance of these conifers did not show a successional pattern and on average both species together contributed only to 3.5% of all individuals in a plot.

### Statistical analyses

We mainly focused on the contribution of interspecific trait variation on trait-enviroment relationships, and thus, used leaf trait values averaged per species. In addition, we tested for an impact of environment on within-species trait variation by modifying the approach of [55]. Intraspecific trait variation was assessed by normalizing trait values within species. All interspecific differences in trait values were eliminated by substracting the species' mean trait value and dividing by the trait's standard deviation. Thus, only species entered the analysis for which more than one trait value had been obtained (106 out of 122) and only traits were used that varied within species (23 out of 26). We then weighted the normalized trait values of all species in a plot by the species' abundances in that plot. The resulting weighted normalized trait values of all CSPs were regressed against all environmental factors that were also used in the Fourth Corner Analysis. All resulting significant relationships were interpreted as environmentally caused intraspecific trait variation.

Interspecific trait variation was related to the environment by using only mean trait values per species. First, the R and Q matrix were submitted to two PCAs, using standardized variables. Second, trait-environment relationships were analysed by Fourth Corner Analysis [56]. This analysis relates the matrix of untransformed environmental variables (R) to a species trait matrix (Q), linked by a matrix of species abundances (L) [3]. The Fourth Corner statistics are derived from comparing the observed distributions of traits of species along the species' positions on the environmental gradient with null models. The result is a matrix of correlations summarizing the significances of the relationship between the set of environmental data and the set of trait data at a given community composition. The R-matrix comprised all environmental data (27 sites×16 variables). In addition to soil variables, the R matrix also contained variables related to structure and to the richness of tree and shrub species in the comparative study plots. Although we included both the set of environmental variables and the set of structure and richness variables in one single analysis, we only consider environmental variables as potential direct causes of trait filtering, while structure and richness con only be considered proxy variables of biotic causes of trait filtering. As richness depends on the number of sampled individuals in the plot, rarefied species richness was calculated [57], based on 100 individuals per plot. The L-matrix comprised the species abundances (27 sites×122 species) and the Q-matrix all traits (122 species×26 traits). Trait-environment relationships were tested for significance by three different null models referred to as model types I, II and IV [41]. In model type I the abundances in matrix L were permuted for each species independently (within each column in the L matrix in Fig. 1c independently of the other columns), thereby testing for non-random associations between sites and environment (L and R matrices) and between species abundances and traits (L and Q matrices, for a scheme see Fig. 2). In model type II the rows (i.e. sites) of table L are permuted, which is equivalent to reshuffling the rows of R and which tests for non-random association between sites and environment (L and R matrices) but keeps the links between L and Q. By keeping this link between species and traits, the obtained significances have to go back to the association between sites and environment. Finally, in model type IV columns (i.e. species) of table L are permuted, thus keeping the association between L and R but not between species abundances and traits (L and Q matrices). As the link between sites and environment is kept, the obtained results have to be ascribed to the connection between species and traits. We would like to point out that the permutation tests are independent of number of traits included in the Q matrix and number of environmental variables in the R matrix because in none of the model types permutation is performed among rows of the Q matrix (i.e. among traits) or among columns in the R matrix (i.e. among environmental variables). In consequence, neither the number of traits or environmental variables nor a potential collinearity among them does affect the results. All three model types were calculated with 9999 permutations. The number of all significant relationships across all traits and environmental variables was assessed by model type for analysing which of the linkages between the matrices was the most important one in explaining the trait-environment relationship. All analyses were computed with the statistical software R (v. 2.10 R Foundation for Statistical Computing) using the ade4 package and the vegan package [58], [59].

## Results

### Environmental relationships

**Figure 3:**
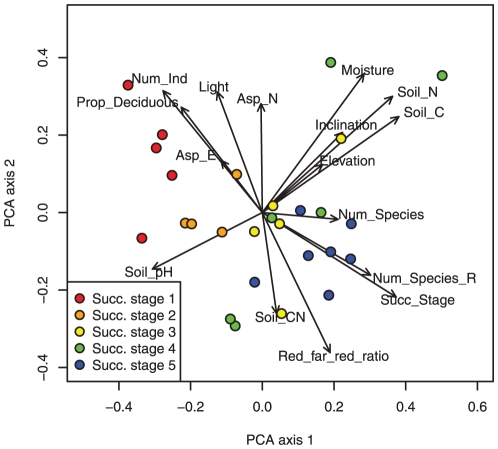
Principal component analysis (PCA) analysing the covariation of all 16environmental variables across all sites (CSPs). Biplot of PCA scores of the first and second axis. The different colours show the successional stages (1: <20 yr, 2: <40 yr, 3: <60 yr, 4: <80 yr, 5: ≥80 yr). Abbreviations of environmental variables: Succ_Stage = Successional stage, Num_Species = Species richness, Num_Species_R = Rarefied species richness, Num_Ind = Number of individuals per plot, Prop_Deciduous = Proportion of deciduous individuals, Soil C = Soil carbon content, Soil N = Soil nitrogen content, Soil CN = Soil C/N ratio, Moisture = Mean soil moisture, Asp_E = Eastness = Sine (aspect), Asp_N = Northness = Cosine (aspect), Light = Mean relative intensity of PAR, Red_far_red_ratio = Mean red∶far-red ratio.

The environmental conditions in the plot were characterized by more than one gradient (Fig. 3). Successional stage was closely and positively related to rarefied species richness, indicating that species number monotonously increased with successional age [14]. An inverse strong relationship to successional stage was encountered to number of individuals, caused by decreasing tree densities with ongoing succession, and to the proportion of deciduous individuals in a plot, because the evergreen trees and shrubs became more abundant with successional time, while the amount of photosynthetically active radiation decreased and the red∶far-red ratio increased. Soil moisture, N and C content as well as pH formed a second gradient that was perpendicular to successional time, while northern and eastern components of aspect, inclination and elevation displayed only weak relationships to other environmental variables (Fig. 3).

### Trait relationships

**Figure 4:**
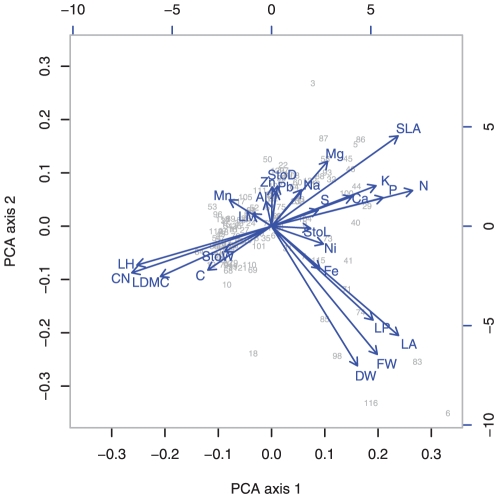
Principal component analysis (PCA) analysing the covariation of all 26 traits across all 122 species. Biplot of PCA scores of the first and second axis. For species codes see Appendix Table S1. Abbreviations of traits: LM = Leaf margin entire, LP = Leaf pinnation, LH = Evergreen leaf habit, FW = Leaf fresh weight, DW = Leaf dry weight, LA = Leaf area, SLA = Specific leaf area, LDMC = Leaf dry matter content, C = Leaf carbon content, N = Leaf nitrogen content, CN = Leaf carbon nitrogen ratio, P = Leaf phosphorous content, K = Leaf potassium content, Ca = Leaf calcium content, Mg = Leaf magnesium content, Al = Leaf aluminium content, Ni = Leaf nickel content, Pb = Leaf lead content, Na = Leaf sodium content, Mn = Leaf manganese content, Zn = Leaf zinc content, Fe = Leaf iron content, S = Leaf sulfur content, StoD = Stomata density, StoL = Stomata length, StoW = Stomata width.

The principal component analysis (PCA) that analysed the covariation of the traits across all species also revealed multiple gradients (Fig. 4). The first PCA axis reflected the gradient of low to high leaf construction costs, with negative loadings for evergreen leaf phenology type, leaf dry matter content and leaf C/N ratio, and positive loadings for the content of N, P, K and Ca. Stomata density was quite unrelated to this axis, but was negatively correlated with stomata width and length, caused by a trade-off between stomata density and stomata size (r = −0.243, p = 0.007). Stomata size was also not correlated with SLA (r = −0.083, p = 0.362). Interestingly, SLA had positive loadings of similar size on both PCA axes in Fig. 4, but was orthogonal to leaf area and leaf fresh and dry weight.

### Trait-environment relationships

The relationship between interspecific trait variation and the environment resulted in 11 significant relationships (Appendix Fig. S1). Within-species variation was positively related between SLA and inclination, LDMC and northern aspects, leaf carbon content and deciduousness, leaf calcium content and light, stomata width and light, as well as between leaf phosphorous content and species richness, rarefied species richness and inclination. In addition, there were negative relationships of leaf manganese content, leaf sulphur content and stomata length to rarefied species number. However, these 11 significances were fewer than would be statistically expected from 368 regression tests (23 traits×16 environmental variables), which, assuming fully randomized data, should at least result in 18 significances at the 5% error level.

**Figure 5:**
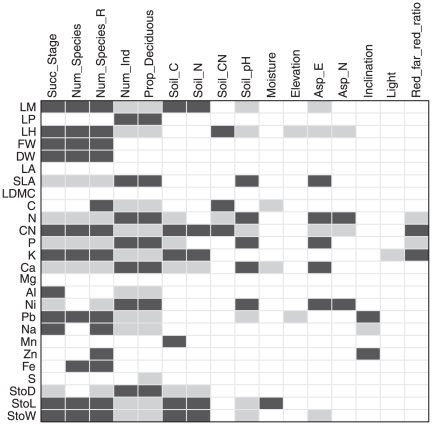
Output of the Fourth Corner Analysis based on model type I. Model type I removes both the link between site and environment and the link between traits and species. Thus, the significant relationships are the result of a combination of the link between species composition and environment as well as the link between species composition and functional traits. Black fields represent significant positive relationships, grey fields significant negative relationships and white fields insignificant relationships. Significance levels are p = 0.05, based on 9999 randomizations. For abbreviations of environmental variables and traits see Fig. 2 and 3, respectively.

In contrast, interspecific trait variation as assessed by the Fourth Corner Analysis revealed strong associations of many traits to the successional gradient (Fig. 5). Most traits with significant relationships to successional stage were also related to species richness and rarefied species number, and in an inverse way to the number of individuals, a finding that was consistent to the results of the correspondence analysis (Fig. 3). Positive relationships of successional age and further variables referring to structure and richness that covaried with age were encountered to evergreen leaf phenology type, size of leaves in terms of weight but not in terms of leaf area, carbon-nitrogen ratio, potassium content and stomata size, seen in increasing stomata length and width. Negative relationships to the previously-mentioned variables were found for specific leaf area and leaf nitrogen, phosphorous and calcium content.

The second main gradient from the correspondence analysis (Fig. 3) was not well reflected in the Fourth Corner Analysis (Fig. 5), with, for example, only three significant trait relationships for soil moisture, one of them showing a negative correlation to leaf calcium content. Although a high C and N content of the soil and a low pH value were significantly correlated with fewer traits than variables associated with successional stage, the significant relationships existed to the same traits, i.e. to the occurrence of entire leaves, high C/N ratio, high potassium and low calcium content and low stomata density. Being less strongly related to other environmental variables in the PCA (Fig. 3), elevation and inclination also displayed only few relationships to traits in the Fourth Corner Analysis (Fig. 5). The incidence of evergreen leaf phenology type decreased with elevation and northeastern aspects, while inclination was positively related to the leaf content of zinc lead, and negatively to sodium.

### Comparing the associations of communities with leaf traits and of species with the environment

A total of 143 significances were obtained for model type I (Fig. 5), as summed up in Table 1 across all traits and all variables of the R matrix. Model type I removed the link between the sites×species matrix (L) and the site×environment matrix (R) as well as between the L matrix and the species×trait matrix (Q). Thus, the significant relationships are the result of a combination of the association of communities with species leaf traits and of species with the environment. A similar magnitude of significant cases (127) was obtained by model type II that only removed the link between sites×species matrix (L) and site×environment matrix (R) (Appendix Fig. S2). In contrast, when removing only the link between sites×species matrix (L) and species×trait matrix (Q) by model type IV, the number of significant relationships was reduced to 75 (Appendix Fig. S3).

**Table 1:** Model comparisons of the Fourth Corner Analysis.

	Model type I	Model type II	Model typeIV
Suc_Sta	17	18	8
Num_Species	14	13	6
Num_Species_R	19	19	7
Num_Ind	17	18	8
Prop_Deciduous	18	21	6
Soil_C	10	8	3
Soil_N	7	7	4
Soil_CN	4	1	2
Soil_pH	11	11	8
Moisture	3	2	1
Elevation	2	1	1
Asp_E	9	1	9
Asp_N	4	0	4
Inclination	3	3	3
Light	1	1	1
Red_far_red_ratio	4	3	6
Sum	143	127	77

Number of significant relationships of all environmental variables obtained by the different permutation models in the Fourth Corner Analysis (see Fig. 2 and Appendix Fig. S2, S3). For abbreviations see Fig. 5.

## Discussion

### Environmental relationships

As postulated in the first hypothesis, most trait-environment relationships were encountered with variables that covaried with the successional gradient. In the course of the succession, many traits that describe the leaf economics spectrum [1], [2] decreased in value, such as specific leaf area and leaf nutrient concentration, while investment in persistence increased, as seen in increasing C/N ratio. Thus, the successional gradient reflected the well-known growth-persistence trade-off [26], [27]. This pattern corresponds to other studies from forests [60], [61] and also grasslands [62], but is contrary to the secondary forest succession from coastal shrubland to mid-successional forests in New Zealand [63], where the authors found a clear shift toward increased leaf palatability and decomposability during succession. However, no abiotic variable had a similarly high number of trait relationships in the Fourth Corner Analysis as successional stage. As hypothesized, factors not related to succession such as elevation, aspect and slope had only a minor impact on trait composition. This low importance of topographical variables forms a contrast to a study on trait distribution in a Chinese tropical forests in Hainan, where slope and aspect have been found to be important predictors [64]. Nevertheless, the few relationships to topographical variables encountered in our study make sense ecologically. The finding of increasing incidence of deciduous leaf phenology with increasing elevation reflects the latitudinal biogeographical gradient, with evergreen leaves dominating in the tropics and deciduous leaves in the temperate zone. The increasing degree of deciduousness with altitude can probably be explained by a higher frost hardiness and higher resistance to snow break of deciduous trees [65]. Similarly, evergreen leaf phenology type was associated with southwestern aspects, which have higher solar elevation angles [66]. Beside a better adaptation of evergreen species to higher temperatures and higher levels of irradiance, the higher sun angles on southwestern slopes might be particularly relevant in the winter time, because then the difference between southern and northern aspects is most pronounced and evergreen species still assimilate carbon [67], [68]. Aspect had also an impact on occurrence of leaves with toothed margins that were predominantly found at eastern aspects. This finding fully complies to the global pattern, where the percentage of toothed leaves was negatively correlated with mean annual temperature, interpreted as a potential adaptation for increased carbon uptake through enhanced sap flow early in the growing season [69].

The low number of significant correlations with inclination is remarkable, given that inclination is related to site factors with a positive impact on species richness [70], [71]. Soil moisture showed surprisingly few trait-relationships, which probably results from our non-continuous moisture measurements. The few encountered relationships displayed the opposite trend than what had been anticipated from the assumption of higher transpiration rates, such as a decrease in leaf calcium content with increasing soil moisture.

### Trait relationships

Traits associated to the leaf economics spectrum turned out to be the most responsive ones, thus confirming the second hypothesis. Most relationships of abiotic variables and of variables related to structure and richness were found for with leaf C to N ratio, which points to a strategy of increasing nutrient conservation and leaf robustness with ongoing succession, but also with decreasing soil pH values [72]. Leaf nitrogen content (LNC) and specific leaf area (SLA) followed the same pattern, but surprisingly and in contrast to [60], leaf dry matter content (LDMC) did not show one significant relationship to any environmental factor. As SLA is determined by both leaf thickness and LDMC [73], subtropical forests seem to respond to environmental gradients mainly by variation in leaf thickness and not by LDMC. Stomata density was found to be unrelated to SLA and leaf nutrient contents. The observed decrease of stomata density with successional time does probably not reflect a gradient in light conditions, as stomata density has been described to be not associated with shade tolerance [34]. Instead, a high stomata density in early-successional stages might allow a more effective and fine-tuned control of conductivity [23]. In contrast, a lower number of stomata per unit area was compensated for by larger stomata sizes in late stages.

We are aware that with our focus on leaf traits we have certainly missed important key traits with well-known functions to tree growth, demography and survival, such as wood density, maximum adult height and seed mass [74]. Leaf traits alone, such as SLA, were found to confer only limited information on the performance of large trees in the tropics [22] and to explain very little of the observed growth–mortality trade-off [74].

### Trait-environment relationships

The numerous and strong relationships of traits to the abiotic variables in this succession series allow the conclusion that environmental filtering is an important mechanism shaping these forest communities. As expected from the third hypothesis, these trait-environment relationships were mainly brought about by interspecific trait variation, while intraspecific trait variation played almost no role at all. Consistent with previous findings that successional time had a large influence on species composition and diversity [14], variables related to richness and forest structure showed strong relationships to even a higher number of traits as compared to those related to the abiotic environment. Assuming that richness and structure, at least partly, reflect biotic filters, brought about by competition, facilitation and complementarity, the biotic component in trait filtering would be stronger than the abiotic component. These arguments give strong support to the suggestion to summarize both abiotic and biotic filtering in the definition of environmental trait filtering [11].

### Comparing the associations of communities with leaf traits and of species with the environment

From the fourth hypothesis we expected that the encountered trait-environment relationships were more based on the association of communities with species leaf traits rather than on the association of species with the environment, which we tested by comparing three different null models with the Fourth Corner Analysis [41]. We found the opposite result, indicating that the impact of variation and position of environmental niche breadths of species was larger than that of trait values within communities. As model type I and II gave almost the same results, most significances can be traced back to the link between species and environment. This finding is consistent to the observed species turnover along the successional gradient [14] and does not support the idea of neutral community assembly [42]. The high importance of the species-environment link sheds some light on the way the performance filters of the community operate [75]. A certain environment will result in eliminating species with traits that confer inadequate local fitness. In a regression framework as suggested by [75] the impact of the environment on the abundance of species will be larger than the impact of eliminated species on trait composition of the community. Thus, it might be that species losses might be buffered by other species with similar traits, lending support to the idea of trait redundancy in the community [76], [77]. Despite the obvious differences of species with respect to important traits (e.g. evergreen vs. deciduous phenology type), trait composition of the different plots was found to be rather homogeneous. This finding is consistent with the null models suggested by us for the same succession series [78], where we described a large equivalence in trait space, i.e. a similar differences between traits, among species. We can add now to this result that also community mean trait values did not change much along the succession series. However, we should point out that the trait composition across all plots was not fully random, because this would not have resulted in significant trait-environment relationships in any null model.

### Conclusion

This first study on trait-environment relationships in Chinese species-rich subtropical forests fully confirmed the existence of environmental filters. Although we were not able to separate abiotic from biotic filtering, the low number of relationships encountered with abiotic environmental variables suggests a strong biotic component in trait filtering. More importantly, applying different null models of the Fourth Corner Analysis we were able to decompose the trait-environment relationship, showing that species were more strongly associated with the environment than traits were associated to the communities. This can be interpreted as general low importance of trait differences for community assembly in this subtropical forest. Yet, the question remains which mechanisms have caused the observed species-environment link. As species composition along the succession series was mainly found to be the result of diffuse immigration [14], future trait studies will have to include dispersal and establishment as key processes that shape the community of this subtropical forest.

## Supporting Information

Figure S1 Relationship between interspecific trait variation and the environment, as obtained by regressing weighted normalized trait values against environmental variables. Black fields represent significant positive relationships, grey fields significant negative relationships and white fields insignificant relationships. Significance levels are p = 0.05. Abbreviations of traits: LM = Leaf margin entire, LP = Leaf pinnation, LH = Evergreen leaf habit, FW = Leaf fresh weight, DW = Leaf dry weight, LA = Leaf area, SLA = Specific leaf area, LDMC = Leaf dry matter content, C = Leaf carbon content, N = Leaf nitrogen content, CN = Leaf carbon nitrogen ratio, P = Leaf phosphorous content, K = Leaf potassium content, Ca = Leaf calcium content, Mg = Leaf magnesium content, Al = Leaf aluminium content, Ni = Leaf nickel content, Pb = Leaf lead content, Na = Leaf sodium content, Mn = Leaf manganese content, Zn = Leaf zinc content, Fe = Leaf iron content, S = Leaf sulfur content, StoD = Stomata density, StoL = Stomata length, StoW = Stomata width. Abbreviations of environmental variables: Succ_Stage = Successional stage, Num_Species = Species richness, Num_Species_R = Rarefied species richness, Num_Ind = Number of individuals per plot, Prop_Decididuous = Proportion of deciduous individuals, Soil C = Soil carbon content, Soil N = Soil nitrogen content, Soil CN = Soil C/N ratio, Moisture = Mean soil moisture, Asp_E = Eastness = Sine (aspect), Asp_N = Northness = Cosine (aspect)), Light = Mean relative intensity of PAR, Red_far_red_ratio = Mean red∶far-red ratio.
(DOC)

Figure S2 Output of the Fourth Corner Analysis based on model type II, removing the link between site and environment; thus, significances result from the association between site and environment. Black fields represent significant positive relationships, grey fields significant negative relationships and white fields insignificant relationships. Significance levels are p = 0.05, based on 9999 randomizations. Abbreviations of traits and environmental variables as in Fig. S1, and additionally LM = Leaf margin entire, LP = Leaf pinnation, LH = Evergreen leaf habit.
(DOC)

Figure S3 Output of the Fourth Corner Analysis based on model type IV, removing the link between species and traits; thus, significances result from the association of species and traits. For details see Fig. S1.
(DOC)

Table S1 Trait values of all species included in the analysis. ID = species code used in Fig. 5. n = number of individuals sampled and analysed. Abbreviations of traits: LM = Leaf margin entire, LP = Leaf pinnation, LH = Evergreen leaf habit, FW = Leaf fresh weight, DW = Leaf dry weight, LA = Leaf area, SLA = Specific leaf area, LDMC = Leaf dry matter content, C = Leaf carbon content, N = Leaf nitrogen content, CN = Leaf carbon nitrogen ratio, P = Leaf phosphorous content, K = Leaf potassium content, Ca = Leaf calcium content, Mg = Leaf magnesium content, Al = Leaf aluminium content, Ni = Leaf nickel content, Pb = Leaf lead content, Na = Leaf sodium content, Mn = Leaf manganese content, Zn = Leaf zinc content, Fe = Leaf iron content, S = Leaf sulfur content, StoD = Stomata density, StoL = Stomata length, StoW = Stomata width.
(DOC)
