# Supplementary material for: Leaf Trait-Environment Relationships in a Subtropical Broadleaved Forest in South-East China
Source: PLoS One. 2012 Apr 23;7(4):e35742. doi: 10.1371/journal.pone.0035742 (PMC3335070; doi:10.1371/journal.pone.0035742)

Figure S1: Relationship between interspecific trait variation and the environment, as obtained by regressing weighted normalized trait values against environmental variables. Black fields represent significant positive relationships, grey fields significant negative relationships and white fields insignificant relationships. Significance levels are p = 0.05. Abbreviations of traits: LM = Leaf margin entire, LP = Leaf pinnation, LH = Evergreen leaf habit, FW = Leaf fresh weight, DW = Leaf dry weight, LA = Leaf area, SLA = Specific leaf area, LDMC = Leaf dry matter content, C = Leaf carbon content, N = Leaf nitrogen content, CN = Leaf carbon nitrogen ratio, P = Leaf phosphorous content, K = Leaf potassium content, Ca = Leaf calcium content, Mg = Leaf magnesium content, Al = Leaf aluminium content, Ni = Leaf nickel content, Pb = Leaf lead content, Na = Leaf sodium content, Mn = Leaf manganese content, Zn = Leaf zinc content, Fe = Leaf iron content, S = Leaf sulfur content, StoD = Stomata density, StoL = Stomata length, StoW = Stomata width.
Abbreviations of environmental variables: Succ_Stage = Successional stage, Num_Species = Species richness, Num_Species_R = Rarefied species richness, Num_Ind = Number of individuals per plot, Prop_Decididuous = Proportion of deciduous individuals, Soil C = Soil carbon content, Soil N = Soil nitrogen content, Soil CN = Soil C/N ratio, Moisture = Mean soil moisture, Asp_E = Eastness =Sine (aspect), Asp_N =Northness = Cosine (aspect) ), Light = Mean relative intensity of PAR, Red_far_red_ratio = Mean red:far-red ratio.


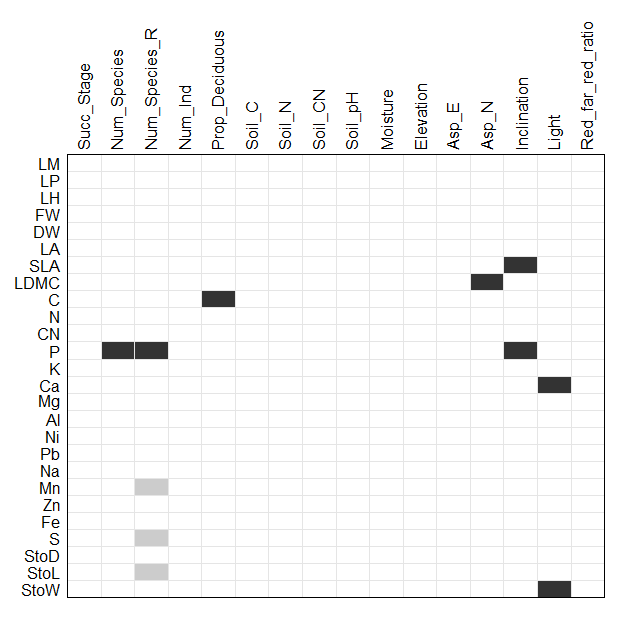

Supplement: Figure S1 — Relationship between interspecific trait variation and the environment, as obtained by regressing weighted normalized trait values against environmental variables. Black fields represent significant positive relationships, grey fields significant negative relationships and white fields insignificant relationships. Significance levels are p = 0.05. Abbreviations of traits: LM = Leaf margin entire, LP = Leaf pinnation, LH = Evergreen leaf habit, FW = Leaf fresh weight, DW = Leaf dry weight, LA = Leaf area, SLA = Specific leaf area, LDMC = Leaf dry matter content, C = Leaf carbon content, N = Leaf nitrogen content, CN = Leaf carbon nitrogen ratio, P = Leaf phosphorous content, K = Leaf potassium content, Ca = Leaf calcium content, Mg = Leaf magnesium content, Al = Leaf aluminium content, Ni = Leaf nickel content, Pb = Leaf lead content, Na = Leaf sodium content, Mn = Leaf manganese content, Zn = Leaf zinc content, Fe = Leaf iron content, S = Leaf sulfur content, StoD = Stomata density, StoL = Stomata length, StoW = Stomata width. Abbreviations of environmental variables: Succ_Stage = Successional stage, Num_Species = Species richness, Num_Species_R = Rarefied species richness, Num_Ind = Number of individuals per plot, Prop_Decididuous = Proportion of deciduous individuals, Soil C = Soil carbon content, Soil N = Soil nitrogen content, Soil CN = Soil C/N ratio, Moisture = Mean soil moisture, Asp_E = Eastness = Sine (aspect), Asp_N = Northness = Cosine (aspect)), Light = Mean relative intensity of PAR, Red_far_red_ratio = Mean red∶far-red ratio. (DOC) [file pone.0035742.s001.doc]
