# Supplementary material for: Leaf Trait-Environment Relationships in a Subtropical Broadleaved Forest in South-East China
Source: PLoS One. 2012 Apr 23;7(4):e35742. doi: 10.1371/journal.pone.0035742 (PMC3335070; doi:10.1371/journal.pone.0035742)

Figure S2: Output of the Fourth Corner Analysis based on model type II, removing the link between site and environment; thus, significances result from the association between site and environment. Black fields represent significant positive relationships, grey fields significant negative relationships and white fields insignificant relationships. Significance levels are p = 0.05, based on 9999 randomizations. Abbreviations of traits and environmental variables as in Fig. S1, and additionally LM = Leaf margin entire, LP = Leaf pinnation, LH = Evergreen leaf habit.


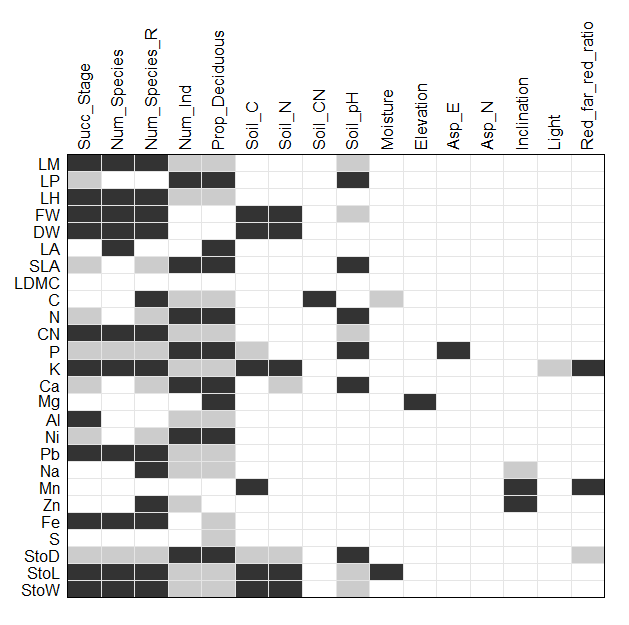

Supplement: Figure S2 — Output of the Fourth Corner Analysis based on model type II, removing the link between site and environment; thus, significances result from the association between site and environment. Black fields represent significant positive relationships, grey fields significant negative relationships and white fields insignificant relationships. Significance levels are p = 0.05, based on 9999 randomizations. Abbreviations of traits and environmental variables as in Fig. S1, and additionally LM = Leaf margin entire, LP = Leaf pinnation, LH = Evergreen leaf habit. (DOC) [file pone.0035742.s002.doc]
