# Supplementary material for: Leaf Trait-Environment Relationships in a Subtropical Broadleaved Forest in South-East China
Source: PLoS One. 2012 Apr 23;7(4):e35742. doi: 10.1371/journal.pone.0035742 (PMC3335070; doi:10.1371/journal.pone.0035742)

Figure S3: Output of the Fourth Corner Analysis based on model type IV, removing the link between species and traits; thus, significances result from the association of species and traits. For details see Fig. S1.


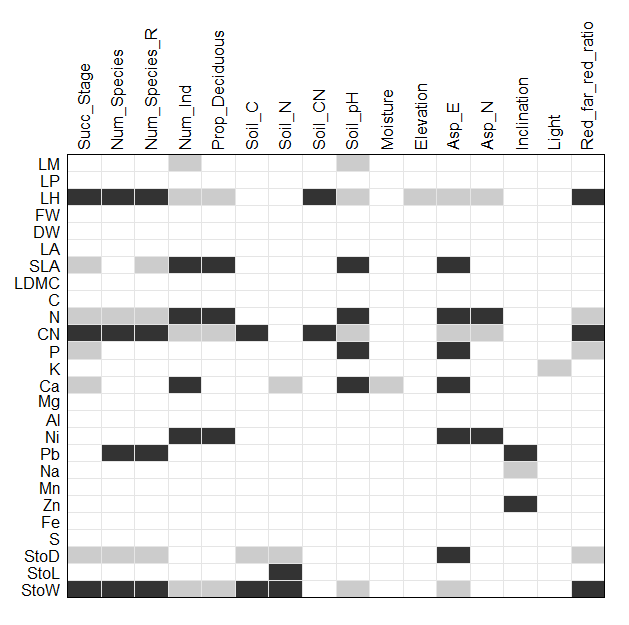

Supplement: Figure S3 — Output of the Fourth Corner Analysis based on model type IV, removing the link between species and traits; thus, significances result from the association of species and traits. For details see Fig. S1. (DOC) [file pone.0035742.s003.doc]
