# Supplementary material for: Leaf Trait-Environment Relationships in a Subtropical Broadleaved Forest in South-East China
Source: PLoS One. 2012 Apr 23;7(4):e35742. doi: 10.1371/journal.pone.0035742 (PMC3335070; doi:10.1371/journal.pone.0035742)
Supplement: Table S1 — Trait values of all species included in the analysis. ID = species code used in Fig. 5. n = number of individuals sampled and analysed. Abbreviations of traits: LM = Leaf margin entire, LP = Leaf pinnation, LH = Evergreen leaf habit, FW = Leaf fresh weight, DW = Leaf dry weight, LA = Leaf area, SLA = Specific leaf area, LDMC = Leaf dry matter content, C = Leaf carbon content, N = Leaf nitrogen content, CN = Leaf carbon nitrogen ratio, P = Leaf phosphorous content, K = Leaf potassium content, Ca = Leaf calcium content, Mg = Leaf magnesium content, Al = Leaf aluminium content, Ni = Leaf nickel content, Pb = Leaf lead content, Na = Leaf sodium content, Mn = Leaf manganese content, Zn = Leaf zinc content, Fe = Leaf iron content, S = Leaf sulfur content, StoD = Stomata density, StoL = Stomata length, StoW = Stomata width. (DOC) [file pone.0035742.s004.doc]

Table S1: Trait values of all species included in the analysis. ID = species code used in Fig. 5. n = number of individuals sampled and analysed. Abbreviations of traits: LM = Leaf margin entire, LP = Leaf pinnation, LH = Evergreen leaf habit, FW = Leaf fresh weight, DW = Leaf dry weight, LA = Leaf area, SLA = Specific leaf area, LDMC = Leaf dry matter content, C = Leaf carbon content, N = Leaf nitrogen content, CN = Leaf carbon nitrogen ratio, P = Leaf phosphorous content, K = Leaf potassium content, Ca = Leaf calcium content, Mg = Leaf magnesium content, Al = Leaf aluminium content, Ni = Leaf nickel content, Pb = Leaf lead content, Na = Leaf sodium content, Mn = Leaf manganese content, Zn = Leaf zinc content, Fe = Leaf iron content, S = Leaf sulfur content, StoD = Stomata density, StoL = Stomata length, StoW = Stomata width.

| ID | Species name | Author | Family | n | LM | LP | LH | FW | DW | LA | SLA | LDMC | C | N | CN | K | Ca | Mg | P | Al | Ni | Pb | Na | Mn | Zn | Fe | S | StoD | StoL | StoW |
| --- | --- | --- | --- | --- | --- | --- | --- | --- | --- | --- | --- | --- | --- | --- | --- | --- | --- | --- | --- | --- | --- | --- | --- | --- | --- | --- | --- | --- | --- | --- |
|  |  |  |  |  |  |  |  | [g] | [mg] | [mm2] | [mm2 mg-1] | [mg  g-1] | [%] [g/g*100] | [%] [g/g*100] | [g/g] | [µg/g] | [µg/g] | [µg/g] | [µg/g] | [µg/g] | [µg/g] | [µg/g] | [µg/g] | [µg/g] | [µg/g] | [µg/g] | [µg/g] | [mm-²] | [µm] | [µm] |
| 1 | *Acer amplum* | Rehder | Sapindaceae | 2 | 0 | 0 | 0 | 5.52 | 2289.50 | 55978 | 26.71 | 404.00 | 45.84 | 1.96 | 23.40 | 15086 | 9356 | 2481 | 485 | 2 | 0 | 4 | 1846 | 1491 | 90 | 202 | 2423 | 30.61 | 19.08 | 13.75 |
| 2 | *Acer cordatum* | Pax | Sapindaceae | 4 | 1 | 0 | 0 | 2.28 | 891.25 | 12423 | 14.42 | 429.94 | 45.71 | 1.48 | 31.77 | 5567 | 11253 | 2108 | 624 | 2 | 0 | 0 | 2091 | 657 | 9 | 82 | 1395 | 38.31 | 21.19 | 14.23 |
| 3 | *Acer pubipalmatum* | Fang | Sapindaceae | 1 | 1 | 0 | 0 | 0.88 | 285.00 | 7601 | 26.67 | 323.13 | 43.59 | 1.76 | 24.81 | 11352 | 15569 | 2389 | 759 | 387 | 0 | 7 | 2709 | 590 | 38 | 213 | 1719 | 490.78 | 22.76 | 14.75 |
| 4 | *Adinandra millettii* | (Hook. et Arn.) Ben. et Hook. | Theaceae | 7 | 0 | 0 | 1 | 4.06 | 1340.43 | 14242 | 10.96 | 333.29 | 44.56 | 1.09 | 41.54 | 11141 | 4463 | 2912 | 402 | 4 | 0 | 1 | 1870 | 1765 | 0 | 99 | 1883 | 22.37 | 34.31 | 28.00 |
| 5 | *Alangium kurzii* | Craib | Alangiaceae | 3 | 0 | 0 | 0 | 6.13 | 1552.67 | 48230 | 26.53 | 299.39 | 45.34 | 3.23 | 15.30 | 15805 | 7095 | 2622 | 993 | 2 | 1 | 11 | 3902 | 579 | 0 | 132 | 2253 | 23.07 | 27.54 | 19.41 |
| 6 | *Albizia kalkora* | (Roxb.) Prain | Fabaceae | 4 | 0 | 1 | 0 | 43.97 | 14597.00 | 255335 | 18.98 | 323.58 | 45.53 | 3.06 | 14.95 | 13404 | 9387 | 3332 | 987 | 2 | 1 | 0 | 1464 | 56 | 0 | 107 | 4160 | 35.32 | 21.84 | 15.97 |
| 7 | *Alniphyllum fortunei* | (Hemsl.) Makino | Styracaeae | 1 | 0 | 0 | 0 | 12.22 | 4011.00 | 63586 | 15.85 | 328.21 | 45.85 | 1.76 | 26.12 | 10339 | 7607 | 1004 | 739 | 2 | 0 | 0 | 0 | 195 | 0 | 75 | 1847 | 29.93 | 28.92 | 21.75 |
| 8 | *Ardisia crenata* | Sims | Myrsinaceae | 3 | 0 | 0 | 1 | 4.11 | 1401.67 | 15971 | 11.52 | 342.33 | 51.46 | 1.58 | 32.37 | 14250 | 10563 | 1462 | 596 | 2 | 0 | 0 | 0 | 209 | 0 | 61 | 901 | 9.64 | 32.34 | 25.70 |
| 9 | *Betula luminifera* | Winkler | Betulaceae | 1 | 1 | 0 | 0 | 8.77 | 3568.00 | 42409 | 11.89 | 406.93 | 38.53 | 1.72 | 22.37 | 11960 | 4989 | 1776 | 775 | 4 | 4 | 1 | 0 | 3317 | 0 | 148 | 6231 | 24.44 | 33.40 | 19.68 |
| 10 | *Camellia chekiang-oleosa* | Hu | Theaceae | 4 | 1 | 0 | 1 | 11.34 | 4367.75 | 26758 | 6.13 | 387.57 | 44.18 | 0.83 | 54.32 | 11436 | 2737 | 1650 | 296 | 4 | 0 | 4 | 0 | 672 | 0 | 122 | 1681 | 13.14 | 29.18 | 26.77 |
| 11 | *Camellia fraterna* | Hance | Theaceae | 6 | 1 | 0 | 1 | 1.82 | 672.67 | 8064 | 11.94 | 370.70 | 44.63 | 1.15 | 39.19 | 8656 | 2471 | 2208 | 504 | 4 | 0 | 0 | 0 | 989 | 0 | 93 | 1982 | 10.87 | 41.32 | 31.86 |
| 12 | *Camellia oleifera* | Abel | Theaceae | 2 | 1 | 0 | 1 | 6.23 | 2294.00 | 15265 | 6.93 | 355.60 | 46.54 | 0.80 | 58.47 | 11703 | 1159 | 1889 | 287 | 4 | 0 | 0 | 0 | 2369 | 0 | 100 | 812 | 13.68 | 31.86 | 25.57 |
| 13 | *Carpinus viminea* | Lindley | Betulaceae | 2 | 1 | 0 | 0 | 1.72 | 686.50 | 11820 | 18.53 | 392.85 | 46.83 | 1.95 | 23.98 | 10843 | 3352 | 1731 | 815 | 3 | 0 | 0 | 0 | 1304 | 32 | 71 | 1347 | 40.63 | 21.99 | 18.68 |
| 14 | *Castanopsis eyrei* | (Champ. ex Benth.) Tutch. | Fagaceae | 2 | 1 | 0 | 0 | 5.09 | 2239.00 | 29447 | 13.15 | 440.64 | 41.72 | 1.53 | 27.37 | 7641 | 3540 | 2471 | 669 | 2 | 0 | 14 | 0 | 1593 | 38 | 65 | 1114 | 49.83 | 26.67 | 20.59 |
| 15 | *Castanopsis carlesii* | (Hemsl.) Hayata | Fagaceae | 3 | 1 | 0 | 1 | 1.63 | 716.33 | 8784 | 13.27 | 433.53 | 48.29 | 1.30 | 37.10 | 7374 | 2397 | 2497 | 408 | 3 | 0 | 1 | 0 | 1312 | 11 | 128 | 1219 | 45.41 | 23.24 | 20.74 |
| 16 | *Castanopsis eyrei* | (Champ. ex Benth.) Tutch. | Fagaceae | 7 | 1 | 0 | 1 | 2.19 | 1046.29 | 9043 | 9.07 | 472.18 | 48.32 | 1.24 | 39.68 | 14108 | 1256 | 1681 | 789 | 3 | 0 | 0 | 1486 | 534 | 6 | 81 | 1393 | 44.79 | 23.11 | 22.44 |
| 17 | *Castanopsis fargesii* | Franchet | Fagaceae | 8 | 1 | 0 | 1 | 3.36 | 2599.25 | 21316 | 8.77 | 466.24 | 47.78 | 1.26 | 38.02 | 6502 | 2089 | 1889 | 406 | 3 | 1 | 2 | 110 | 1988 | 15 | 90 | 1294 | 29.63 | 24.06 | 21.27 |
| 18 | *Castanopsis tibetana* | Hance | Fagaceae | 2 | 1 | 0 | 1 | 22.00 | 10523.50 | 85516 | 8.17 | 481.59 | 47.10 | 1.01 | 47.04 | 6508 | 1050 | 1982 | 339 | 4 | 1 | 5 | 307 | 608 | 3 | 143 | 1029 | 48.54 | 22.99 | 20.78 |
| 19 | *Chimonanthus salicifolius* | S. Y. Hu | Calycanthaceae | 2 | 0 | 0 | 1 | 1.76 | 566.50 | 11669 | 20.82 | 316.51 | 42.24 | 1.74 | 24.65 | 6521 | 8550 | 2026 | 537 | 2 | 2 | 0 | 0 | 1100 | 56 | 124 | 1259 | 26.55 | 30.08 | 28.92 |
| 20 | *Cinnamomum subavenium* | Miquel | Lauraceae | 7 | 0 | 0 | 1 | 2.98 | 1490.14 | 12531 | 8.59 | 498.81 | 48.55 | 0.99 | 50.81 | 5837 | 2347 | 1052 | 878 | 2 | 0 | 0 | 398 | 469 | 0 | 105 | 876 | 40.98 | 19.96 | 14.70 |
| 21 | *Cleyera japonica* | Thunb. | Theaceae | 5 | 0 | 0 | 1 | 6.05 | 2536.60 | 18637 | 8.02 | 412.96 | 44.55 | 0.79 | 58.61 | 8342 | 1336 | 3293 | 498 | 1 | 0 | 0 | 0 | 1163 | 0 | 37 | 2402 | 14.83 | 30.94 | 23.51 |
| 22 | *Cornus kousa* | Buerger | Cornaceae | 2 | 1 | 0 | 0 | 2.43 | 677.00 | 13853 | 21.36 | 309.82 | 44.90 | 1.17 | 38.24 | 12109 | 9963 | 3265 | 368 | 3 | 0 | 13 | 1446 | 24 | 11 | 122 | 3858 | 9.51 | 35.02 | 22.80 |
| 23 | *Corylopsis glandulifera* | Hemsley | Hamamelidaceae | 4 | 1 | 0 | 0 | 5.63 | 1877.25 | 34101 | 19.11 | 329.92 | 45.13 | 1.64 | 27.71 | 6844 | 11198 | 2771 | 694 | 2 | 0 | 2 | 797 | 60 | 0 | 76 | 1629 | 16.95 | 26.89 | 19.91 |
| 24 | *Cyclobalanopsis glauca* | (Thunb.) Oers. | Fagaceae | 6 | 1 | 0 | 1 | 4.93 | 2334.83 | 23707 | 10.46 | 472.06 | 48.31 | 1.31 | 37.23 | 6469 | 6941 | 2529 | 879 | 2 | 0 | 5 | 1651 | 358 | 35 | 66 | 1592 | 53.89 | 20.96 | 16.05 |
| 25 | *Cyclobalanopsis gracilis* | (Re. et Wi.) Cheng et Hong | Fagaceae | 3 | 1 | 0 | 1 | 2.76 | 1281.33 | 13711 | 11.58 | 457.37 | 48.29 | 1.63 | 29.69 | 6479 | 2203 | 1741 | 823 | 2 | 0 | 0 | 935 | 1798 | 7 | 87 | 1605 | 66.53 | 20.93 | 12.47 |
| 26 | *Cyclobalanopsis myrsinaefolia* | Oerst. | Fagaceae | 2 | 1 | 0 | 1 | 2.58 | 1319.00 | 14606 | 11.00 | 522.81 | 46.89 | 1.44 | 32.54 | 7565 | 3753 | 2573 | 499 | 3 | 1 | 0 | 1127 | 811 | 9 | 356 | 1279 | 57.92 | 18.92 | 13.57 |
| 27 | *Cyclobalanopsis nubium* | (Hand.-Mazz.) Chun | Fagaceae | 2 | 0 | 0 | 1 | 3.26 | 1338.00 | 14299 | 10.82 | 412.18 | 48.07 | 1.52 | 31.57 | 8626 | 4246 | 1587 | 593 | 2 | 0 | 0 | 607 | 697 | 0 | 51 | 1131 | 64.79 | 21.12 | 17.33 |
| 28 | *Cyclobalanopsis stewardiana* | (Camus) Hsu et Jen | Fagaceae | 2 | 1 | 0 | 1 | 4.02 | 2187.50 | 16483 | 7.55 | 540.59 | 50.68 | 1.42 | 35.70 | 5944 | 734 | 1008 | 479 | 2 | 0 | 0 | 1286 | 1134 | 1 | 75 | 1521 | 58.90 | 22.75 | 16.07 |
| 29 | *Dalbergia hupeana* | Hance | Fabaceae | 2 | 0 | 1 | 0 | 6.30 | 2273.50 | 55565 | 25.48 | 362.98 | 47.26 | 3.47 | 13.62 | 19969 | 428 | 4141 | 957 | 2 | 2 | 0 | 865 | 562 | 68 | 90 | 3010 | 37.46 | 19.73 | 12.17 |
| 30 | *Daphniphyllum oldhamii* | Hemsley | Daphniphyllaceae | 5 | 0 | 0 | 1 | 7.19 | 2487.40 | 28779 | 11.64 | 352.56 | 46.05 | 1.26 | 36.83 | 12168 | 3393 | 4759 | 475 | 3 | 0 | 0 | 0 | 16 | 0 | 75 | 1325 | 32.89 | 22.46 | 17.69 |
| 31 | *Dendropanax dentiger* | (Harms) Merr. | Araliaceae | 3 | 0 | 0 | 1 | 16.44 | 4568.00 | 65964 | 15.08 | 288.76 | 46.22 | 1.12 | 41.49 | 16188 | 5466 | 2742 | 727 | 2 | 1 | 0 | 899 | 1671 | 289 | 303 | 1519 | 19.03 | 29.89 | 22.48 |
| 32 | *Diospyros kaki* | Thunb. | Ebenaceae | 2 | 0 | 0 | 0 | 8.68 | 1888.50 | 49695 | 26.18 | 219.80 | 43.96 | 1.18 | 37.15 | 49816 | 6794 | 3161 | 457 | 2 | 1 | 0 | 621 | 2296 | 3 | 129 | 1765 | 18.06 | 34.03 | 18.45 |
| 33 | *Elaeocarpus chinensis* | (Gard. et Champ.) Hook. | Elaeocarpaceae | 2 | 0 | 0 | 1 | 1.43 | 563.00 | 8624 | 15.15 | 395.02 | 46.49 | 1.43 | 32.65 | 6144 | 1230 | 1082 | 558 | 2 | 2 | 0 | 109 | 2008 | 0 | 124 | 2000 | 22.55 | 22.17 | 17.17 |
| 34 | *Elaeocarpus decipiens* | Hemsley | Elaeocarpaceae | 4 | 1 | 0 | 1 | 5.00 | 1883.50 | 21748 | 12.32 | 368.07 | 46.65 | 1.21 | 38.67 | 10363 | 3229 | 1364 | 438 | 2 | 2 | 0 | 0 | 973 | 0 | 41 | 1704 | 35.00 | 22.55 | 15.45 |
| 35 | *Elaeocarpus japonicus* | Sieb. et Zucc. | Elaeocarpaceae | 3 | 1 | 0 | 1 | 6.63 | 2570.00 | 30965 | 15.31 | 374.32 | 46.89 | 1.58 | 30.29 | 10197 | 4104 | 1872 | 704 | 2 | 0 | 0 | 0 | 621 | 0 | 56 | 3249 | 23.12 | 26.45 | 21.81 |
| 36 | *Euonymus centidens* | Léveillé | Celastraceae | 2 | 1 | 0 | 0 | 0.97 | 328.50 | 4535 | 17.83 | 316.27 | 46.59 | 1.36 | 34.24 | 11231 | 5433 | 2713 | 695 | 2 | 0 | 0 | 0 | 24 | 0 | 62 | 4943 | 13.86 | 28.45 | 23.74 |
| 37 | *Eurya alata* | Kobuski | Theaceae | 2 | 1 | 0 | 1 | 1.43 | 572.00 | 6318 | 11.67 | 394.48 | 43.38 | 1.04 | 42.42 | 7330 | 695 | 3036 | 548 | 4 | 0 | 0 | 0 | 2185 | 0 | 88 | 2062 | 26.74 | 28.70 | 28.47 |
| 38 | *Eurya muricata* | Dunn | Theaceae | 8 | 1 | 0 | 1 | 4.26 | 1650.13 | 13443 | 8.36 | 387.42 | 43.47 | 0.92 | 48.43 | 7704 | 1064 | 2567 | 362 | 4 | 0 | 0 | 0 | 658 | 0 | 35 | 1377 | 24.58 | 25.90 | 22.74 |
| 39 | *Eurya rubiginosa* | Chang | Theaceae | 5 | 1 | 0 | 1 | 2.82 | 1057.20 | 11876 | 11.36 | 372.97 | 43.16 | 0.94 | 46.88 | 8421 | 902 | 3158 | 501 | 4 | 0 | 0 | 654 | 1916 | 0 | 167 | 2178 | 35.02 | 22.02 | 18.84 |
| 40 | *Euscaphis japonica* | (Thunb.) Kanitz | Staphyleaceae | 3 | 1 | 1 | 0 | 9.18 | 2471.67 | 45129 | 19.88 | 269.21 | 43.24 | 2.28 | 19.05 | 20246 | 8504 | 3800 | 623 | 3 | 5 | 0 | 826 | 51 | 9 | 80 | 2667 | 25.46 | 25.97 | 16.38 |
| 41 | *Fraxinus insularis* | Hemsley | Oleaceae | 4 | 1 | 1 | 0 | 13.15 | 3956.75 | 74294 | 21.30 | 295.46 | 45.94 | 1.91 | 24.21 | 17078 | 8376 | 3800 | 556 | 2 | 2 | 0 | 467 | 41 | 7 | 88 | 3317 | 16.15 | 30.93 | 20.32 |
| 42 | *Gardenia jasminoides* | Ellis | Rubiaceae | 2 | 0 | 0 | 1 | 5.79 | 1724.00 | 27049 | 15.66 | 305.64 | 42.85 | 1.40 | 30.94 | 18208 | 6183 | 2013 | 426 | 2 | 0 | 0 | 21 | 86 | 24 | 145 | 1478 | 32.68 | 24.45 | 14.29 |
| 43 | *Glochidion puberum* | (Linn.) Hutch. | Euphorbiaceae | 2 | 0 | 0 | 0 | 8.33 | 2719.00 | 41979 | 21.76 | 301.01 | 44.74 | 1.19 | 37.54 | 16425 | 8029 | 4339 | 511 | 2 | 2 | 0 | 558 | 1868 | 45 | 104 | 2378 | 40.55 | 20.44 | 13.98 |
| 44 | *Hovenia trichocarpa* | Chun et Tsiang | Rhamnaceae | 2 | 1 | 0 | 0 | 9.94 | 2919.00 | 89424 | 35.38 | 280.53 | 44.91 | 2.53 | 17.75 | 25789 | 9482 | 2377 | 630 | 2 | 1 | 0 | 308 | 1311 | 42 | 87 | 1737 | 28.33 | 23.30 | 14.99 |
| 45 | *Hydrangea chinensis* | Maxim. | Saxifragaceae | 4 | 1 | 0 | 0 | 7.99 | 1306.75 | 34618 | 28.82 | 161.69 | 39.10 | 2.44 | 16.34 | 17147 | 1199 | 4047 | 758 | 4 | 3 | 0 | 628 | 484 | 31 | 195 | 1996 | 8.87 | 38.09 | 22.27 |
| 46 | *Hydrangea paniculata* | Siebold | Saxifragaceae | 1 | 1 | 0 | 0 | 9.64 | 2211.00 | 43871 | 19.84 | 229.45 | 43.36 | 1.66 | 26.20 | 17471 | 18515 | 7372 | 970 | 3 | 7 | 0 | 145 | 235 | 41 | 91 | 1663 | 30.97 | 29.23 | 19.92 |
| 47 | *Ilex buergeri* | Miquel | Aquifoliaceae | 4 | 1 | 0 | 1 | 2.44 | 1019.75 | 11276 | 11.11 | 419.73 | 48.04 | 1.09 | 45.21 | 9521 | 4440 | 2963 | 330 | 3 | 1 | 0 | 337 | 2792 | 8 | 389 | 1235 | 21.05 | 24.84 | 20.78 |
| 48 | *Ilex elmerrilliana* | S. Y. Hu | Aquifoliaceae | 2 | 0 | 0 | 1 | 3.22 | 1298.33 | 11281 | 8.87 | 394.38 | 47.31 | 0.85 | 55.71 | 12300 | 4960 | 3471 | 459 | 2 | 0 | 0 | 0 | 1284 | 108 | 23 | 1699 | 22.52 | 29.29 | 23.53 |
| 49 | *Ilex litseifolia* | Hu et Tang | Aquifoliaceae | 2 | 1 | 0 | 1 | 4.25 | 1927.50 | 17708 | 9.65 | 443.90 | 47.59 | 0.86 | 55.56 | 7355 | 6859 | 2903 | 619 | 2 | 0 | 0 | 136 | 1685 | 194 | 43 | 1011 | 35.60 | 25.68 | 22.45 |
| 50 | *Ilex pubescens* | Hook. et Arn. | Aquifoliaceae | 2 | 1 | 0 | 1 | 0.66 | 189.00 | 4044 | 21.09 | 288.29 | 45.94 | 1.42 | 32.26 | 17835 | 802 | 4938 | 446 | 3 | 5 | 1 | 0 | 1931 | 246 | 177 | 2018 | 31.45 | 25.33 | 20.32 |
| 51 | *Ilex purpurea* | Miquel | Aquifoliaceae | 5 | 1 | 0 | 1 | 2.50 | 998.60 | 12991 | 12.73 | 397.84 | 48.34 | 1.07 | 48.57 | 13049 | 2982 | 3209 | 409 | 2 | 1 | 0 | 25 | 1267 | 35 | 49 | 1811 | 33.45 | 26.00 | 21.24 |
| 52 | *Ilex rotunda* | Thunb. | Aquifoliaceae | 6 | 1 | 0 | 1 | 3.58 | 1254.50 | 15025 | 12.74 | 351.17 | 47.95 | 1.28 | 38.23 | 19081 | 2457 | 5577 | 520 | 3 | 0 | 0 | 0 | 929 | 40 | 52 | 1585 | 20.24 | 32.03 | 26.02 |
| 53 | *Ilex suaveolens* | (Léve.) Loes. | Aquifoliaceae | 2 | 1 | 0 | 1 | 2.49 | 987.00 | 12015 | 12.08 | 406.20 | 48.81 | 0.80 | 61.08 | 14491 | 6060 | 3615 | 306 | 406 | 1 | 0 | 575 | 5064 | 100 | 100 | 1399 | 26.03 | 31.92 | 25.07 |
| 54 | *Ilex wilsonii* | Loes. | Aquifoliaceae | 4 | 0 | 0 | 1 | 1.70 | 783.75 | 5565 | 7.16 | 460.19 | 50.00 | 1.14 | 43.93 | 6812 | 5625 | 2216 | 565 | 3 | 0 | 0 | 134 | 2385 | 88 | 47 | 1609 | 26.21 | 34.67 | 27.79 |
| 55 | *Itea chinensis* | Hook. et Arno. | Saxifragaceae | 4 | 1 | 0 | 1 | 4.69 | 1763.25 | 22929 | 13.31 | 375.40 | 45.82 | 1.13 | 40.74 | 4986 | 8280 | 2513 | 538 | 2 | 0 | 2 | 0 | 2067 | 71 | 46 | 1318 | 48.35 | 18.62 | 16.87 |
| 56 | *Laurocerasus spinulosa* | (Sieb. et Zucc.) Schneid. | Rosaceae | 1 | 1 | 0 | 0 | 4.95 | 2231.00 | 16611 | 7.45 | 451.16 | 49.61 | 0.97 | 51.16 | 8689 | 5922 | 1749 | 452 | 217 | 2 | 0 | 195 | 15 | 5 | 42 | 1253 | 48.77 | 18.64 | 12.42 |
| 57 | *Lindera aggregata* | (Sims) Kesterm. | Lauraceae | 2 | 0 | 0 | 1 | 1.52 | 751.00 | 9284 | 12.71 | 487.42 | 50.36 | 1.34 | 37.51 | 8172 | 5064 | 1422 | 333 | 2 | 0 | 0 | 0 | 927 | 25 | 92 | 718 | 37.95 | 20.33 | 18.54 |
| 58 | *Lindera erythrocarpa* | Makino | Lauraceae | 1 | 0 | 0 | 0 | 2.69 | 656.00 | 17127 | 26.11 | 243.78 | 48.30 | 2.20 | 21.97 | 12742 | 2801 | 1402 | 1625 | 3 | 0 | 1 | 0 | 1951 | 99 | 137 | 4508 | 24.82 | 26.22 | 17.54 |
| 59 | *Lindera glauca* | (Sieb. et Zucc.) Bl. | Lauraceae | 3 | 0 | 0 | 0 | 3.69 | 1656.33 | 22620 | 13.99 | 446.29 | 48.82 | 1.90 | 25.82 | 9758 | 5105 | 658 | 809 | 2 | 0 | 0 | 349 | 1207 | 94 | 144 | 3100 | 32.62 | 25.71 | 13.45 |
| 60 | *Lindera reflexa* | Hemsley | Lauraceae | 3 | 0 | 0 | 0 | 2.68 | 803.00 | 22830 | 29.59 | 304.60 | 47.79 | 1.81 | 27.17 | 15722 | 4438 | 1920 | 623 | 3 | 1 | 0 | 55 | 1385 | 23 | 291 | 1866 | 18.73 | 23.21 | 15.78 |
| 61 | *Liquidambar formosana* | Hance | Hamamelidaceae | 3 | 1 | 0 | 0 | 7.69 | 3051.33 | 47410 | 17.16 | 386.40 | 44.68 | 1.31 | 34.08 | 7027 | 7333 | 2785 | 445 | 3 | 2 | 0 | 0 | 879 | 0 | 62 | 782 | 29.02 | 19.61 | 15.55 |
| 62 | *Lithocarpus glaber* | (Thunb.) Nakai | Fagaceae | 7 | 0 | 0 | 1 | 4.88 | 2292.29 | 21019 | 9.26 | 474.13 | 49.86 | 1.28 | 39.58 | 6879 | 5227 | 1259 | 378 | 2 | 4 | 3 | 0 | 1399 | 0 | 130 | 1010 | 36.54 | 25.12 | 20.59 |
| 63 | *Litsea coreana* | Léveillé | Lauraceae | 6 | 0 | 0 | 1 | 1.94 | 897.67 | 10010 | 11.14 | 463.28 | 49.26 | 1.23 | 40.35 | 8844 | 5883 | 948 | 606 | 3 | 0 | 0 | 0 | 829 | 14 | 84 | 1056 | 28.14 | 23.61 | 20.34 |
| 64 | *Litsea cubeba* | (Lour.) Pers. | Lauraceae | 4 | 0 | 0 | 0 | 2.05 | 671.75 | 14019 | 20.57 | 340.68 | 49.53 | 2.14 | 25.22 | 9571 | 6059 | 2911 | 854 | 2 | 0 | 0 | 0 | 923 | 30 | 95 | 1702 | 21.65 | 34.82 | 17.74 |
| 65 | *Litsea elongata* | Hook. | Lauraceae | 3 | 1 | 0 | 1 | 5.29 | 1687.00 | 19977 | 12.61 | 371.46 | 49.36 | 1.37 | 36.00 | 8715 | 4728 | 1805 | 378 | 3 | 0 | 1 | 0 | 622 | 9 | 178 | 1112 | 25.63 | 22.26 | 17.43 |
| 66 | *Loropetalum chinense* | (R. Br.) Oliv. | Hamamelidaceae | 7 | 0 | 0 | 1 | 0.85 | 344.14 | 4804 | 14.93 | 403.43 | 45.53 | 1.27 | 36.50 | 4897 | 14154 | 1395 | 636 | 3 | 1 | 1 | 0 | 33 | 0 | 105 | 1100 | 36.06 | 23.05 | 17.60 |
| 67 | *Lyonia ovalifolia* | (Wall.) Drude | Ericaceae | 3 | 0 | 0 | 0 | 3.89 | 1592.00 | 29856 | 18.94 | 411.58 | 48.56 | 1.71 | 28.62 | 9396 | 7332 | 2057 | 555 | 2 | 2 | 3 | 0 | 5536 | 0 | 108 | 1181 | 25.21 | 31.47 | 20.52 |
| 68 | *Machilus grijsii* | Hance | Lauraceae | 4 | 0 | 0 | 1 | 6.34 | 2777.00 | 29792 | 10.68 | 441.59 | 49.15 | 0.94 | 52.55 | 11614 | 3260 | 1180 | 347 | 2 | 7 | 3 | 0 | 1519 | 20 | 233 | 665 | 34.97 | 21.11 | 14.08 |
| 69 | *Machilus pauhoi* | Kanehira | Lauraceae | 2 | 0 | 0 | 1 | 7.40 | 3122.50 | 32521 | 11.30 | 414.18 | 49.65 | 1.37 | 36.71 | 7578 | 5006 | 1095 | 538 | 2 | 9 | 1 | 0 | 701 | 0 | 103 | 1214 | 28.71 | 20.57 | 14.85 |
| 70 | *Machilus thunbergii* | Sieb. et Zucc. | Lauraceae | 8 | 0 | 0 | 1 | 4.27 | 1799.50 | 17154 | 9.74 | 426.15 | 48.40 | 1.04 | 47.65 | 9494 | 4798 | 1162 | 301 | 2 | 5 | 0 | 0 | 541 | 0 | 79 | 1100 | 28.99 | 24.68 | 22.07 |
| 71 | *Magnolia cylindrica* | Wilson | Magnoliaceae | 2 | 0 | 0 | 0 | 24.21 | 7110.00 | 104849 | 14.28 | 309.93 | 44.14 | 2.11 | 21.45 | 11199 | 13881 | 2397 | 630 | 2 | 1 | 0 | 0 | 908 | 0 | 79 | 1428 | 15.22 | 37.47 | 16.44 |
| 72 | *Malus leiocalyca* | Huang | Rosaceae | 2 | 1 | 0 | 0 | 3.57 | 1547.50 | 21182 | 13.64 | 436.62 | 47.49 | 1.49 | 31.83 | 9727 | 10599 | 2591 | 431 | 2 | 3 | 0 | 0 | 20 | 0 | 170 | 1112 | 32.48 | 26.16 | 17.99 |
| 73 | *Meliosma flexuosa* | Pamp. | Sabiaceae | 3 | 1 | 0 | 0 | 13.12 | 4177.33 | 88537 | 23.74 | 312.30 | 44.29 | 1.63 | 27.22 | 14747 | 9933 | 2975 | 612 | 2 | 3 | 0 | 0 | 988 | 0 | 116 | 2176 | 14.03 | 37.17 | 20.34 |
| 74 | *Meliosma oldhamii* | Miquel | Sabiaceae | 6 | 1 | 1 | 0 | 21.34 | 5732.83 | 101150 | 18.68 | 266.08 | 44.22 | 1.72 | 25.84 | 18615 | 7241 | 2717 | 652 | 2 | 7 | 0 | 30 | 495 | 0 | 51 | 1987 | 6.72 | 46.64 | 30.90 |
| 75 | *Michelia skinneriana* | Dunn | Magnoliaceae | 1 | 0 | 0 | 1 | 3.54 | 1186.00 | 14642 | 12.35 | 334.84 | 44.78 | 2.22 | 20.20 | 7062 | 4461 | 2382 | 820 | 2 | 2 | 0 | 0 | 831 | 0 | 32 | 1293 | 22.68 | 24.08 | 11.02 |
| 76 | *Myrica rubra* | Sieb. et Zucc. | Myricaceae | 8 | 1 | 0 | 1 | 3.40 | 1441.25 | 16251 | 11.60 | 423.48 | 48.39 | 1.44 | 33.98 | 8117 | 4634 | 1711 | 317 | 2 | 1 | 0 | 0 | 439 | 10 | 108 | 1706 | 49.61 | 19.40 | 14.68 |
| 77 | *Neolitsea aurata* | (Hay.) Koid. | Lauraceae | 5 | 0 | 0 | 1 | 2.16 | 1141.60 | 12346 | 10.80 | 524.92 | 48.95 | 1.16 | 42.46 | 7222 | 4673 | 1042 | 440 | 2 | 2 | 0 | 545 | 1994 | 32 | 253 | 981 | 41.74 | 19.47 | 15.39 |
| 78 | *Nyssa sinensis* | Oliver | Nyssaceae | 1 | 0 | 0 | 0 | 6.16 | 2421.00 | 38127 | 15.75 | 393.34 | 46.43 | 0.91 | 50.86 | 9902 | 5550 | 2765 | 824 | 621 | 17 | 0 | 97 | 2593 | 0 | 69 | 1594 | 146.72 | 31.03 | 21.41 |
| 79 | *Osmanthus cooperi* | Hemsley | Oleaceae | 2 | 0 | 0 | 1 | 5.61 | 2528.50 | 20807 | 7.87 | 455.51 | 48.59 | 1.21 | 40.47 | 7056 | 7345 | 1866 | 485 | 2 | 1 | 0 | 0 | 8 | 4 | 27 | 1202 | 60.27 | 27.66 | 23.80 |
| 80 | *Photinia beauverdiana* | Schneid. | Rosaceae | 2 | 1 | 0 | 0 | 1.43 | 521.00 | 11759 | 25.69 | 378.90 | 47.68 | 1.50 | 31.87 | 20082 | 6148 | 2974 | 583 | 2 | 2 | 0 | 0 | 902 | 79 | 140 | 1616 | 18.33 | 28.92 | 13.56 |
| 81 | *Photinia hirsuta* | Hand.-Mazz. | Rosaceae | 2 | 1 | 0 | 0 | 1.33 | 567.50 | 9796 | 17.40 | 424.19 | 48.51 | 1.09 | 44.70 | 14154 | 6925 | 2809 | 495 | 2 | 2 | 0 | 163 | 68 | 13 | 101 | 1038 | 12.43 | 22.75 | 19.38 |
| 82 | *Photinia parvifolia* | (Prit.) Schneid. | Rosaceae | 2 | 1 | 0 | 0 | 1.32 | 576.00 | 12317 | 22.62 | 430.66 | 48.66 | 1.64 | 29.70 | 11923 | 5014 | 3189 | 620 | 2 | 4 | 0 | 0 | 1242 | 38 | 110 | 1260 | 6.76 | 37.02 | 27.63 |
| 83 | *Picrasma quassioides* | (Don) Benn. | Simaroubaceae | 1 | 1 | 1 | 0 | 21.46 | 6735.00 | 143122 | 21.25 | 313.84 | 44.20 | 2.17 | 20.35 | 17173 | 9294 | 1826 | 989 | 2 | 13 | 0 | 0 | 168 | 0 | 9552 | 3082 | 24.16 | 26.09 | 16.04 |
| 84 | *Pieris formosa* | (Wall.) Don | Ericaceae | 1 | 1 | 0 | 1 | 2.84 | 1281.50 | 10326 | 8.47 | 453.92 | 49.05 | 0.91 | 54.32 | 4429 | 6501 | 800 | 366 | 2 | 1 | 0 | 0 | 3654 | 31 | 69 | 1166 | 62.61 | 23.58 | 22.34 |
| 85 | *Platycarya strobilacea* | Sieb. et Zucc. | Juglandaceae | 2 | 1 | 1 | 0 | 14.69 | 6326.50 | 92431 | 14.53 | 429.50 | 45.40 | 1.61 | 28.51 | 8069 | 7433 | 1441 | 814 | 2 | 4 | 0 | 0 | 312 | 0 | 74 | 976 | 52.74 | 26.84 | 16.00 |
| 86 | *Premna microphylla* | Turc. | Verbenaceae | 2 | 0 | 0 | 0 | 3.62 | 862.00 | 28542 | 33.57 | 237.85 | 42.76 | 2.82 | 15.18 | 30905 | 6036 | 3372 | 1012 | 2 | 3 | 0 | 0 | 1142 | 2 | 135 | 1344 | 17.34 | 27.07 | 17.17 |
| 87 | *Prunus discoides* | Yu et Li | Rosaceae | 1 | 1 | 0 | 0 | 1.20 | 441.00 | 11684 | 26.49 | 366.58 | 45.55 | 2.29 | 19.88 | 17854 | 11501 | 5541 | 844 | 2 | 3 | 0 | 0 | 106 | 0 | 146 | 1172 | 107.22 | 19.40 | 11.67 |
| 88 | *Prunus schneideriana* | Koehne | Rosaceae | 2 | 1 | 0 | 0 | 3.97 | 1420.50 | 27649 | 19.45 | 357.49 | 45.23 | 2.09 | 21.61 | 21847 | 9379 | 3484 | 1018 | 2 | 3 | 0 | 27 | 80 | 0 | 98 | 1291 | 63.67 | 18.00 | 13.38 |
| 89 | *Quercus phillyreoides* | Gray | Fagaceae | 4 | 1 | 0 | 1 | 1.16 | 602.25 | 4624 | 7.83 | 511.51 | 48.30 | 1.21 | 40.04 | 6204 | 6798 | 1245 | 367 | 2 | 2 | 0 | 0 | 316 | 0 | 199 | 806 | 50.27 | 23.83 | 20.11 |
| 90 | *Quercus serrata* | Murray | Fagaceae | 4 | 0 | 0 | 0 | 3.75 | 1673.25 | 19523 | 12.11 | 453.44 | 47.33 | 1.85 | 25.96 | 7548 | 6262 | 1687 | 743 | 2 | 5 | 0 | 158 | 753 | 0 | 56 | 1264 | 62.36 | 21.78 | 15.79 |
| 91 | *Randia cochinchinensis* | (Lour.) Merr. | Rubiaceae | 2 | 0 | 0 | 1 | 4.16 | 1925.00 | 16786 | 8.71 | 463.63 | 49.24 | 1.71 | 28.87 | 12036 | 4454 | 2224 | 734 | 2 | 4 | 0 | 188 | 986 | 0 | 30 | 1234 | 40.94 | 20.06 | 18.63 |
| 92 | *Rhaphiolepis indica* | (Linn.) Lindl. | Rosaceae | 5 | 1 | 0 | 1 | 1.95 | 813.00 | 7705 | 9.85 | 411.57 | 46.49 | 1.19 | 39.58 | 11902 | 9156 | 4454 | 703 | 2 | 4 | 0 | 73 | 217 | 42 | 58 | 960 | 28.07 | 25.59 | 20.09 |
| 93 | *Rhamnus crenata* | Sieb. et Zucc. | Rhamnaceae | 1 | 0 | 0 | 0 | 2.89 | 970.00 | 23667 | 24.40 | 335.18 | 43.89 | 2.59 | 16.94 | 17281 | 10025 | 2944 | 547 | 2 | 3 | 0 | 0 | 82 | 0 | 125 | 2400 | 11.71 | 33.74 | 13.68 |
| 94 | *Rhododendron latouchea* | Franchet | Ericaceae | 7 | 0 | 0 | 1 | 6.40 | 2430.00 | 19797 | 8.19 | 383.57 | 47.26 | 0.91 | 53.96 | 6657 | 8356 | 2602 | 352 | 3 | 2 | 0 | 33 | 358 | 0 | 62 | 526 | 22.83 | 29.11 | 24.10 |
| 95 | *Rhododendron mariesii* | Hemsl. et Wils. | Ericaceae | 2 | 0 | 0 | 0 | 1.34 | 391.50 | 8177 | 21.23 | 293.86 | 44.78 | 1.31 | 34.12 | 11585 | 6896 | 3788 | 526 | 3 | 6 | 0 | 0 | 3274 | 10 | 137 | 1311 | 30.01 | 31.33 | 19.06 |
| 96 | *Rhododendron ovatum* | (Lindl.) Planch. | Ericaceae | 7 | 0 | 0 | 1 | 2.19 | 926.00 | 10535 | 11.51 | 425.74 | 46.13 | 0.95 | 49.36 | 6850 | 10810 | 3469 | 270 | 3 | 2 | 5 | 0 | 6949 | 0 | 148 | 950 | 21.15 | 27.18 | 22.87 |
| 97 | *Rhododendron simsii* | Planch. | Ericaceae | 5 | 0 | 0 | 0 | 1.17 | 409.00 | 8583 | 23.67 | 340.67 | 44.40 | 1.60 | 27.93 | 5805 | 9821 | 4564 | 416 | 2 | 3 | 0 | 0 | 2763 | 3 | 79 | 1288 | 17.50 | 26.26 | 15.82 |
| 98 | *Rhus hypoleuca* | Champ. ex Benth. | Anacardiaceae | 2 | 0 | 1 | 0 | 25.51 | 8120.50 | 113431 | 14.47 | 311.67 | 48.35 | 1.36 | 35.67 | 16836 | 4808 | 1968 | 619 | 2 | 0 | 0 | 0 | 54 | 0 | 62 | 2100 | 31.81 | 31.46 | 21.39 |
| 99 | *Schima superba* | Gardn. et Champ. | Theaceae | 7 | 1 | 0 | 1 | 7.05 | 2966.57 | 26666 | 9.16 | 420.41 | 49.12 | 1.24 | 41.07 | 7110 | 5049 | 1983 | 376 | 3 | 3 | 1 | 0 | 1396 | 0 | 59 | 1447 | 27.07 | 27.27 | 24.24 |
| 100 | *Schoepfia jasminodora* | Sieb. et Zucc. | Olacaceae | 1 | 0 | 0 | 0 | 8.11 | 2857.00 | 30481 | 10.67 | 352.28 | 42.50 | 2.46 | 17.25 | 21889 | 19964 | 2826 | 1081 | 3 | 7 | 0 | 0 | 2271 | 7 | 71 | 3895 | 33.92 | 29.05 | 17.77 |
| 101 | *Sorbus dunnii* | Rehder | Rosaceae | 1 | 1 | 0 | 0 | 5.57 | 2854.00 | 32627 | 11.43 | 512.30 | 48.01 | 1.44 | 33.34 | 5954 | 9614 | 1844 | 594 | 2 | 2 | 1 | 313 | 1341 | 5 | 139 | 1027 | 37.98 | 28.36 | 20.72 |
| 102 | *Sorbus folgneri* | (Schneid.) Rehd. | Rosaceae | 2 | 1 | 0 | 0 | 4.41 | 1906.50 | 35331 | 20.41 | 445.45 | 46.61 | 1.73 | 26.91 | 13681 | 10235 | 4593 | 841 | 2 | 1 | 0 | 0 | 30 | 0 | 64 | 1079 | 23.66 | 28.35 | 23.15 |
| 103 | *Styrax dasyanthus* | Perkins | Styracaeae | 3 | 0 | 0 | 0 | 4.57 | 1636.00 | 31053 | 18.96 | 398.30 | 48.61 | 1.59 | 30.77 | 8704 | 6320 | 1368 | 601 | 2 | 2 | 0 | 0 | 462 | 0 | 67 | 1424 | 24.95 | 27.57 | 15.28 |
| 104 | *Styrax odoratissima* | Champ. | Styracaeae | 6 | 0 | 0 | 0 | 6.89 | 2407.00 | 51214 | 22.25 | 346.95 | 46.17 | 2.01 | 23.28 | 8655 | 7073 | 1732 | 673 | 2 | 2 | 0 | 0 | 1617 | 0 | 70 | 2013 | 22.24 | 27.96 | 16.23 |
| 105 | *Symplocos anomala* | Diels | Symplocaceae | 3 | 1 | 0 | 1 | 1.70 | 585.33 | 7734 | 13.42 | 342.47 | 37.82 | 1.12 | 34.16 | 5150 | 1161 | 2963 | 254 | 4 | 3 | 0 | 0 | 4580 | 0 | 124 | 8694 | 18.13 | 27.00 | 23.98 |
| 106 | *Symplocos heishanensis* | Hayata | Symplocaceae | 2 | 0 | 0 | 1 | 6.04 | 1925.00 | 27463 | 14.51 | 323.55 | 41.95 | 1.46 | 28.76 | 14855 | 1797 | 1867 | 488 | 4 | 2 | 0 | 0 | 12 | 0 | 40 | 4381 | 23.08 | 28.11 | 21.27 |
| 107 | *Symplocos olongifolia* | Fletch. | Symplocaceae | 2 | 0 | 0 | 1 | 1.86 | 520.50 | 7600 | 14.92 | 281.67 | 35.29 | 1.19 | 29.58 | 4647 | 10371 | 1671 | 405 | 4 | 2 | 0 | 0 | 798 | 0 | 62 | 7277 | 29.01 | 19.06 | 10.64 |
| 108 | *Symplocos paniculata* | (Thunb.) Miq. | Symplocaceae | 1 | 1 | 0 | 0 | 4.50 | 1247.00 | 22181 | 17.79 | 277.30 | 47.75 | 1.64 | 29.17 | 10197 | 10871 | 2422 | 791 | 2 | 4 | 0 | 0 | 2828 | 313 | 80 | 1413 | 13.52 | 29.74 | 18.69 |
| 109 | *Symplocos setchuensis* | (Thunb.) Sieb. et Zucc. | Symplocaceae | 3 | 1 | 0 | 1 | 4.39 | 1973.67 | 15327 | 7.92 | 448.91 | 41.84 | 1.02 | 41.00 | 6545 | 1683 | 1814 | 308 | 4 | 3 | 0 | 56 | 887 | 0 | 37 | 3664 | 18.64 | 22.22 | 19.15 |
| 110 | *Symplocos stellaris* | Diels | Symplocaceae | 7 | 0 | 0 | 1 | 10.20 | 3413.57 | 26800 | 7.82 | 337.57 | 45.41 | 1.19 | 38.97 | 6524 | 318 | 2207 | 373 | 4 | 2 | 0 | 57 | 1146 | 0 | 56 | 5464 | 20.08 | 26.68 | 19.82 |
| 111 | *Symplocos sumuntia* | Buch.-Ham. | Symplocaceae | 6 | 1 | 0 | 1 | 1.72 | 556.17 | 6521 | 13.43 | 320.21 | 37.33 | 1.24 | 30.24 | 4636 | 5705 | 2483 | 326 | 5 | 2 | 0 | 314 | 2092 | 0 | 147 | 7920 | 26.79 | 21.12 | 17.94 |
| 112 | *Syzygium buxifolium* | Hook. et Arno. | Myrtaceae | 6 | 0 | 0 | 1 | 0.90 | 394.33 | 3842 | 9.92 | 435.29 | 49.00 | 0.94 | 52.75 | 7765 | 6025 | 2262 | 421 | 2 | 1 | 0 | 1406 | 342 | 0 | 0 | 1176 | 30.75 | 20.39 | 19.62 |
| 113 | *Tarenna mollissima* | Hook. et Arn. | Rubiaceae | 2 | 0 | 0 | 0 | 3.32 | 1201.50 | 24375 | 20.27 | 363.15 | 43.27 | 1.99 | 22.85 | 18681 | 10970 | 2553 | 522 | 3 | 4 | 0 | 1435 | 118 | 0 | 111 | 3486 | 18.64 | 30.26 | 21.80 |
| 114 | *Ternstroemia gymnanthera* | (Wight et Arn.) Bedd. | Theaceae | 4 | 0 | 0 | 1 | 4.10 | 1627.50 | 14444 | 8.92 | 394.95 | 47.11 | 0.95 | 50.78 | 11571 | 6864 | 2933 | 192 | 3 | 2 | 0 | 880 | 59 | 0 | 8 | 1876 | 19.54 | 28.33 | 22.74 |
| 115 | *Tilia endochrysea* | Hand.-Mazz. | Tiliaceae | 2 | 1 | 0 | 0 | 16.36 | 5241.50 | 77283 | 14.28 | 353.51 | 48.80 | 1.89 | 25.85 | 15129 | 7342 | 2737 | 873 | 2 | 6 | 0 | 0 | 1987 | 0 | 90 | 1635 | 66.33 | 19.81 | 16.15 |
| 116 | *Toxicodendron succedaneum* | (Linn.) Kuntze | Anacardiaceae | 2 | 0 | 1 | 0 | 35.32 | 13742.50 | 144081 | 14.24 | 354.91 | 45.96 | 1.58 | 29.23 | 12320 | 12210 | 1930 | 675 | 2 | 2 | 0 | 0 | 1192 | 0 | 54 | 2093 | 34.51 | 26.48 | 17.31 |
| 117 | *Vaccinium bracteatum* | Thunb. | Ericaceae | 5 | 1 | 0 | 1 | 1.49 | 513.50 | 7610 | 15.00 | 342.34 | 46.39 | 1.23 | 38.32 | 5978 | 8857 | 2511 | 346 | 3 | 2 | 1 | 0 | 3964 | 15 | 125 | 1922 | 31.15 | 20.08 | 14.24 |
| 118 | *Vaccinium carlesii* | Dunn | Ericaceae | 9 | 1 | 0 | 1 | 0.60 | 209.22 | 2638 | 12.75 | 351.92 | 47.42 | 1.06 | 44.98 | 3065 | 5899 | 2203 | 349 | 2 | 1 | 1 | 22 | 924 | 0 | 98 | 1461 | 29.11 | 21.00 | 16.84 |
| 119 | *Vaccinium mandarinorum* | Diels | Ericaceae | 7 | 1 | 0 | 1 | 3.01 | 1024.29 | 11265 | 11.19 | 342.55 | 47.13 | 0.92 | 51.53 | 2419 | 4052 | 1850 | 259 | 2 | 5 | 0 | 0 | 944 | 0 | 138 | 1180 | 23.92 | 24.18 | 17.67 |
| 120 | *Viburnum erosum* | Thunb. | Caprifoliaceae | 3 | 1 | 0 | 0 | 2.16 | 800.33 | 15893 | 21.78 | 362.23 | 45.61 | 1.43 | 31.92 | 10735 | 8383 | 4392 | 551 | 2 | 0 | 5 | 0 | 1430 | 93 | 102 | 1059 | 30.81 | 26.58 | 21.99 |
| 121 | *Viburnum sempervirens* | K. Koch | Caprifoliaceae | 1 | 1 | 0 | 1 | 8.49 | 3030.00 | 34369 | 11.34 | 356.89 | 49.60 | 1.14 | 43.52 | 6434 | 5785 | 2409 | 403 | 2 | 1 | 0 | 0 | 856 | 11 | 124 | 903 | 19.99 | 36.10 | 26.71 |
| 122 | *Weigela japonica* | Thunb. | Caprifoliaceae | 2 | 1 | 0 | 0 | 4.77 | 1362.50 | 30963 | 24.42 | 279.37 | 44.53 | 1.39 | 33.14 | 23553 | 7716 | 3907 | 599 | 3 | 1 | 0 | 0 | 233 | 9 | 74 | 3300 | 25.54 | 34.38 | 20.37 |
